# Lomerizine inhibits LPS-mediated neuroinflammation and tau hyperphosphorylation by modulating NLRP3, DYRK1A, and GSK3α/β

**DOI:** 10.3389/fimmu.2023.1150940

**Published:** 2023-06-26

**Authors:** Jin-Hee Park, Jeong-Woo Hwang, Hyun-ju Lee, Geum Mi Jang, Yoo Joo Jeong, Joonho Cho, Jinsoo Seo, Hyang-Sook Hoe

**Affiliations:** ^1^ Department of Neural Development and Disease, Korea Brain Research Institute (KBRI), Daegu, Republic of Korea; ^2^ Department of Brain Sciences, Daegu Gyeongbuk Institute of Science & Technology, Daegu, Republic of Korea

**Keywords:** lomerizine, neuroinflammation, tauopathy, Alzheimer’s disease, DYRK1A, NLRP3

## Abstract

**Introduction:**

Lomerizine is a calcium channel blocker that crosses the blood–brain barrier and is used clinically in the treatment of migraines. However, whether lomerizine is beneficial in modulating neuroinflammatory responses has not been tested yet.

**Methods:**

To assess the potential of lomerizine for repurposing as a treatment for neuroinflammation, we investigated the effects of lomerizine on LPS-induced proinflammatory responses in BV2 microglial cells, Alzheimer’s disease (AD) excitatory neurons differentiated from induced pluripotent stem cells (iPSCs), and in LPS-treated wild type mice.

**Results:**

In BV2 microglial cells, lomerizine pretreatment significantly reduced LPS-evoked proinflammatory cytokine and NLRP3 mRNA levels. Similarly, lomerizine pretreatment significantly suppressed the increases in Iba-1, GFAP, proinflammatory cytokine and NLRP3 expression induced by LPS in wild-type mice. In addition, lomerizine posttreatment significantly decreased LPS-stimulated proinflammatory cytokine and SOD2 mRNA levels in BV2 microglial cells and/or wild-type mice. In LPS-treated wild-type mice and AD excitatory neurons differentiated from iPSCs, lomerizine pretreatment ameliorated tau hyperphosphorylation. Finally, lomerizine abolished the LPS-mediated activation of GSK3α/β and upregulation of DYRK1A, which is responsible for tau hyperphosphorylation, in wild-type mice.

**Discussion:**

These data suggest that lomerizine attenuates LPS-mediated neuroinflammatory responses and tau hyperphosphorylation and is a potential drug for neuroinflammation- or tauopathy-associated diseases.

## Introduction

Multiple studies have identified direct or indirect links between neuroinflammation and neurodegenerative diseases, including Alzheimer’s disease (AD). For instance, in AD patients, microglial expression of the proinflammatory cytokine IL-1β increases significantly after β-amyloid (Aβ) plaque formation (1, 2), and expression of translocation protein 18 kDa (TPSO), a biomarker of neuroinflammation, is increased in activated glial cells (3). In addition, intense localization of microglia and astrocytes near senile plaques is observed in AD patients, suggesting that these plaques contribute to AD pathology (4). This clinical evidence notwithstanding, no drug regulating neuroinflammation has been proven to prevent or treat AD.

Tau hyperphosphorylation is closely associated with AD pathoprogression. Under normal conditions, tau stabilizes microtubules by bundling them, but under pathological conditions, tau is hyperphosphorylated and dissociates from microtubules, leading to microtubule disassembly. Several studies have found that tau hyperphosphorylation and the formation of neurofibrillary tangles (NFTs), aggregates of hyperphosphorylated tau, are associated with neuroinflammation (5). For example, in AD patients, activated IL-1α-immunoreactive microglia and S100β-immunoreactive astrocytes induce NFT formation in the parahippocampal cortex, resulting in neuronal degeneration (6). Tau hyperphosphorylation leads to microglial activation and neuroinflammation, which are essential for AD pathogenesis and progression (7). These observations suggest that inhibiting tau hyperphosphorylation and neuroinflammatory responses may be a therapeutic strategy for AD.

Lomerizine is an antagonist of L- and T-type voltage-gated calcium channels that is prescribed to migraine patients and is known to cross the blood-brain barrier (8, 9). Interestingly, lomerizine exhibits anti-colorectal cancer (CRC) effects by suppressing the proliferation and progression of human CRC cells (10). In addition, several recent studies have reported neuroprotective effects of lomerizine, including reduction of retinal damage in a rat ischemia model and reduced prevalence of stroke in patients with leukoencephalopathy (11, 12). However, no study has examined whether lomerizine modulates pathological features associated with neurodegeneration, namely, neuroinflammatory responses and tau hyperphosphorylation in the brain.

To address this gap and provide evidence supporting the repurposing of lomerizine as a therapy for neuroinflammation, the present study investigated the effects of lomerizine on neuroinflammatory responses induced by the endotoxin lipopolysaccharide (LPS) *in vitro* and *in vivo*. Lomerizine pretreatment significantly attenuated LPS-evoked proinflammatory cytokine levels and NLRP3 mRNA levels in BV2 microglial cells. In addition, lomerizine pretreatment significantly reduced LPS-induced microgliosis, astrogliosis, proinflammatory cytokine upregulation, and LPS-primed NLRP3 expression in wild-type mice. Moreover, lomerizine posttreatment markedly inhibited LPS-stimulated proinflammatory cytokine levels in BV2 microglial cells and wild-type mice. Finally, lomerizine significantly inhibited tau phosphorylation in LPS-induced wild-type mice and AD neurons differentiated from induced pluripotent stem cells (iPSCs), potentially by reducing the LPS-stimulated activation of GSK3α/β and DYRK1A. These data indicate that lomerizine has beneficial effects on neuroinflammation and tau hyperphosphorylation *in vivo*.

## Materials and methods

### Ethics statement

All experimental procedures were approved by the institutional biosafety committee (IBC) and performed in accordance with approved animal protocols of the Korea Brain Research Institute (KBRI, approval no. IACUC-20-00061).

### Lomerizine

**Figure 1:**
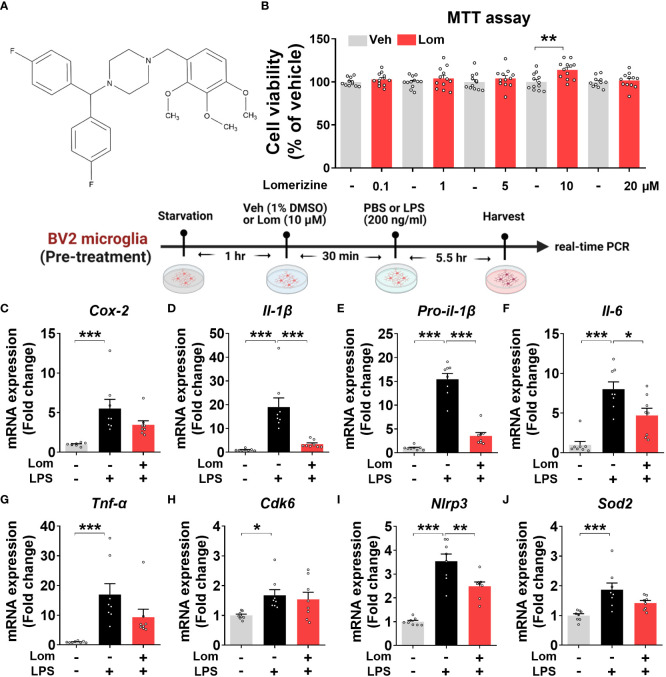
Lomerizine pretreatment downregulates LPS-evoked proinflammatory cytokine levels in BV2 microglial cells. **(A)** Stucture of lomerizine. **(B)** MTT assays were conducted after treating BV2 microglial cells with vehicle (0.1-0.5% DMSO) or lomerizine (0.1, 1, 5, 10, or 20 µM**)** (n = 11-12/group). **(C–J)** BV2 microglial cells were pretreated with vehicle (1% DMSO) or lomerizine (10 µM) for 30 min and then treated with LPS (200 ng/ml) or PBS for 5.5 h (n = 8/group). Then, real-time PCR analysis of proinflammatory cytokine mRNA levels **(C–G)** and CDK6, NLRP3, and SOD2 mRNA levels was performed **(H–J)**. *p < 0.05, **p < 0.01, ***p < 0.001.

Lomerizine (L5751, LKT Labs, St. Paul, MN, USA) was dissolved in 1% DMSO and used at a dose of 10 µM in *in vitro* assays (**Figure 1A**). For *in vivo* assays, lomerizine was intraperitoneally (i.p.) administered at a dose of 20 mg/kg or 30 mg/kg dissolved in 2% DMSO.

### Cell culture

The microglial cell line BV2 (kindly provided by Dr. Kyung-Ho Suk) was cultured in high-glucose Dulbecco’s modified Eagle medium (DMEM, LM 001-01, WELGENE Inc., Gyeongsangbuk-do, Republic of Korea) with 5% fetal bovine serum (SH30919.03, HyClone, Cytiva, Marlborough, Massachusetts, USA) in a 5% CO_2_ incubator.

### MTT

The viability of BV2 microglial cells was assessed by the 3-(4,5-dimethylthiazol-2-yl)-2,5-diphenyltetrazolium bromide (MTT; VWR Chemicals, Solon, OH, USA) assay. To evaluate the cytotoxicity of lomerizine *in vitro*, BV2 microglial cells were exposed to vehicle (0.1%–0.5%) or lomerizine (0.1, 1, 5, 10, or 20 µM) for 24 h. The cells were then incubated with MTT solution for 3 h, and the formazan product was dissolved in DMSO for 20 min and quantified by measuring the absorbance at 570 nm in a SPECTROstar Nano microplate reader (BMG Labtech, Ortenberg, Germany).

### Real-time PCR

In the *in vitro* studies, the effects of lomerizine pretreatment on LPS-induced proinflammatory cytokine levels were investigated by treating BV2 microglial cells with lomerizine (10 µM) or vehicle (1% DMSO) for 30 min followed by LPS (200 ng/ml, L2630, *Escherichia coli*, Sigma–Aldrich, St. Louis, MO, USA) or PBS for 5.5 h. The effects of lomerizine posttreatment were evaluated in BV2 microglial cells treated with LPS (200 ng/ml) or PBS for 30 min and lomerizine (10 µM) or vehicle (1% DMSO) for 5.5 h.

In the *in vivo* studies, the effects of lomerizine alone on proinflammatory cytokine levels were evaluated in wild-type mice treated with vehicle (2% DMSO) or lomerizine (30 mg/kg, i.p.) daily for 7 days. The effects of lomerizine posttreatment on LPS-mediated proinflammatory cytokine levels were investigated in wild-type mice injected with LPS (10 mg/kg) or PBS followed 30 min later by three injections of lomerizine (30 mg/kg) or vehicle (2% DMSO) at 2.5-h intervals. The mice were sacrificed 2.5 h after the final injection.

RNA was extracted from cells or brain tissue using TRIzol (Invitrogen, Waltham, MA, USA) (13) and used to synthesize cDNA with Superscript cDNA Premix Kit II (GeNetBio, SR-5000, Daejeon, Republic of Korea). The cDNA was used in 40-cycle real-time PCR with Fast SYBR Green Master Mix (Thermo Fisher Scientific, Waltham, MA, USA) in a QuantStudio™ 5 system (Thermo Fisher Scientific, Waltham, MA, USA). The cycle threshold (Ct) value for GAPDH was used for normalization. **Table 1** presents the primer sequences.

**Table 1:** Sequences of primers used for real-time PCR.

Gene		Sequence
IL-6	Forward Reverse	5’-CCA CGG CCT TCC CTA CTT C-3’ 5’-TTG GGA GTG GTA TCC TCT GTG A-3’
pro-IL-1β	Forward Reverse	5’-CCA CGG CCT TCC CTA CTT C-3’ 5’-TTG GGA GTG GTA TCC TCT GTG A-3’
IL-1β	Forward Reverse	5’-TTG ACG GAC CCA AAA AGA TG-3’ 5’-AGG ACA GCC CAG GTC AAA G-3’
NLRP3	Forward Reverse	5’-TCC ACA ATT CTG ACC CAC AA-3’ 5’-ACC TCA CAG AGG GTC ACC AC-3’
COX-2	Forward Reverse	5’CCA CTT CAA GGG AGT CTG GA-3’ 5’-AGT CAT CTG CTA CGG GAG GA-3’
TNF-α	Forward Reverse	5’- TCC AGG CGG TGC CTA TGT-3’ 5’-GCC CCT GCC ACA AGC A-3’
CDK6	Forward Reverse	5’-CTC CAC AGA GTA GTG CAT CGT-3’ 5’-CGA GGT AAG GGC CAT CTG AAA A-3’
SOD2	Forward Reverse	5’-GGC CAA GGG AGA TGT TAC AA-3’ 5’-GAA CCT TGG ACT CCC ACA-3’
GAPDH	Forward Reverse	5’-TGG GCT ACA CTG AGG ACC ACT-3’ 5’-GGG AGT GTC TGT TGA AGT CG-3’

### Human AD iPSCs

The AD iPSC line was generated from healthy iPSCs (GM23720, Coriell Institute, NJ, USA) by introducing the APPswe mutation (APP KM670/671NL) by CRISPR/Cas9 genome editing with sgRNA (5’-GCAGAATTCCGACATGACTC-3’) and ssODN (5’- GTTCTGGGTTGACAAATATCAAGACGGAGGAGATCTCTGAAGTGAATCTCGATGCAGAATTCCGACATGACTCAGGATATGAAGTTCATCATCAAAAATTG-3’). The iPSCs were fed mTeSR1 medium (StemCell Technologies, Vancouver, Canada) daily. For excitatory neuronal differentiation, the iPSCs were transferred to a 6-well plate at a density of 4x10^6^ cells/well with 4 ml of mTeSR1 media with Rock inhibitor (Y-27632, 20 μM, Tocris, Bristol, UK). Ninety minutes later, the lentivirus with rtTA and the Ngn2-GFP-expressing vector were added, and the cells were mixed vigorously. After 18 h, the culture medium was fully replaced with neuronal differentiation medium (NDM) [DMEM/F12 (Gibco, Waltham, MA, USA), N2 (1x, Gibco, Waltham, MA, USA), MEM non-essential amino acid solution (NEAA)] with 10 ng/ml BDNF (Peprotech, Cranbury, NJ, USA), 10 ng/ml NT-3 (Peprotech, Cranbury, NJ, USA), 0.2 μg/ml laminin (Corning, Corning, NY, USA), and 2 μg/ml doxycycline (Sigma Aldrich, St. Louis, Missouri, USA) to induce Ngn2 expression. Two days later, the medium was fully replaced with fresh NDM containing B27 (1x, Gibco, Waltham, MA, USA) and GlutaMAX (1x, Gibco, Waltham, MA, USA) instead of N2 and NEAA. After 2 days, the cells were seeded in a Matrigel (Corning, Corning, NY, USA)-coated 24-well plate at a density of 1.5x10^5^/well. The following day, the medium was replaced with human iPSC (the same iPSC line used for neuronal differentiation)-derived astrocyte-conditioned medium (ACM) containing 1 μM Ara-C (Sigma Aldrich, St. Louis, Missouri, USA). Three days later, the medium was replaced with ACM containing 10 ng/ml BDNF, 10 ng/ml NT-3, 0.2 μg/ml laminin, and 0.5 μg/ml doxycycline. Half of the medium volume was replaced with fresh medium every 4 days, and the cells were used for experiments at 4 weeks of differentiation.

### Immunofluorescence staining of iPSCs

The AD iPSC-derived neurons were washed with cold PBS and fixed with 4% paraformaldehyde (PFA) at room temperature for 15 min. Next, the neurons were washed three times with PBS, incubated for 1 h in blocking solution [10% normal donkey serum (Merck, Darmstadt, Germany), 2% BSA (Gemini Bio, CA, USA), 1 M glycine (Sigma Aldrich, St. Louis, Missouri, USA) in PBST (PBS + 0.1% Triton-X 100)], and incubated for 1 h with the primary antibody at room temperature. The cells were then treated with the secondary antibody diluted in blocking solution for 1 h at room temperature. After washing with PBST, the cells were incubated with Hoechst 33342 (Sigma Aldrich) for nuclear staining and mounted for imaging. Fluorescence microscopy images of the immunostained cells were obtained by an LSM 800 (Zeiss, Jena, Germany). The primary and secondary antibodies are listed in **Table 2**.

**Table 2:** Antibodies used in this study.

Primary antibodies					
Immunogen	Host	Dilution	Manufacturer	Cat. no	Application
Iba-1	Rabbit	1:500	Wako	019-19741	IF
GFAP	Chicken	1:500	Millipore	Ab5541	IF
IL-6	Mouse	1:100	Santa Cruz	SC-57315	IF
IL-1β	Rabbit	1:200	Abcam	Ab9722	IF
NLRP3	Rabbit	1:200-1:1000	Novus	NBP2-12446	IF/WB
AT8	Mouse	1:100-1:200	Invitrogen	Mn1020	IF/ICC
AT100	Mouse	1:200	Invitrogen	Mn1020	IF
AT180	Mouse	1:100	Invitrogen	Mn1020	IF
p-Tau (Thr181)	Rabbit	1:200	Cell Signaling	#12885	ICC
p-Tau (Ser396)	Mouse	1:200	Cell Signaling	#9632	ICC
p-GSK3α/β	Rabbit	1:200-1:1000	Abcam	Ab75745	IF/WB
DYRK1A	Rabbit	1:200	Abcam	Ab180910	IF
β-actin	mouse	1:5000	Santa Cruz	SC-47778	WB
Secondary antibodies					
**Antibody**		**Dilution**	**Manufacturer**	**Cat. no**	**Application**
Goat anti-mouse IgG, 488		1:100-1:200	Invitrogen	A11001	IF
Goat anti-rabbit IgG, 594		1:200	Invitrogen	A11012	IF
Goat anti-rabbit IgG, 555		1:100-1:200	Invitrogen	A21428	IF
Goat anti-chicken IgG, 488		1:200	Invitrogen	A11039	IF
Donkey anti-mouse IgG, 594		1:200	Jackson ImmunoResearch	715-585-150	ICC
Donkey anti-rabbit IgG, 594		1:200	Jackson ImmunoResearch	711-585-152	ICC
Goat anti-rabbit, HRP		1:10000	Enzo	ADI-SAB-300-J	WB
Goat anti-mouse, HRP		1:10000	Enzo	ADI-SAB-100-J	WB

### Wild-type mice

Male eight-week-old C57BL6/N mice (Orient-Bio Company) were housed in a pathogen-free facility environmentally controlled at 22°C under a 12-h photoperiod. The mice had free access to food and water during the experiment.

### Treatment of wild-type mice with lomerizine

To investigate whether lomerizine directly modulates LPS-induced neuroinflammation in the central nervous system (CNS), wild-type mice (8 weeks old) were injected with lomerizine (30 mg/kg, i.p.) or vehicle (2% DMSO) daily for 14 consecutive days. Two hours after the injection on day 14, LPS (3 µg in 1 µl of saline/ventricle) or saline was administered *via* bilateral intracerebroventricular (i.c.v.) injection.

For bilateral i.c.v. injection, the mouse was anesthetized with 2,2,2-tribromoethanol (Sigma–Aldrich, St. Louis, MO, USA, 2.5% v/v, 150 mg/kg, i.p.), placed in the stereotaxic apparatus (Stoelting, Wood Dale, IL, USA), and fixed with blunt ear bars. Then, a scalp incision was made, a hole was drilled, and a needle connected to a 10-μl syringe (Hamilton, Reno, NV, USA) was inserted into the lateral ventricle (LV). The stereotaxic coordinates for the LV were −0.5 mm anterior/posterior, ± 1.0 mm medial/lateral, and -2.25 mm dorsal/ventral from the bregma. LPS or saline was injected at a flow rate of 0.3 μl/min. Four hours after the LPS injection, the cortex and hippocampus were dissected and stored at -80°C until analysis.

#### Lomerizine pretreatment

To investigate whether lomerizine modulates LPS-induced neuroinflammation *in vivo*, lomerizine (20 or 30 mg/kg, i.p.) or vehicle (2% DMSO) was administered to wild-type mice (8 weeks old) daily for 7 days. On day 7, LPS (10 mg/kg, i.p.) or PBS was administered 30 min after the lomerizine or vehicle injection. Eight hours later, the mice were sacrificed, perfused with PBS, and fixed in 4% paraformaldehyde (PFA). The brains were postfixed in 4% paraformaldehyde (PFA) overnight at 4°C. After removing the 4% PFA, the brains were stored in 30% sucrose for 72 h at 4°C, frozen at -80°C with OCT compound (Sakura Finetek USA, Torrance, CA), and sliced at a thickness of 30 µm at -23°C using a Leica CM1850 cryostat (Leica Biosystems, Buffalo Grove, IL, USA).

#### Lomerizine posttreatment

To test whether lomerizine posttreatment modulates LPS-evoked neuroinflammatory responses *in vivo*, wild-type mice (8 weeks old) were injected with LPS (10 mg/kg, i.p.) or PBS. Thirty minutes after the LPS injection, lomerizine (30 mg/kg, i.p.) or vehicle (2% DMSO) was administered three times at intervals of 2.5 h. The mice were sacrificed 2.5 h after the final lomerizine or vehicle injection, and the cortex and hippocampus were dissected and stored at -80°C until analysis.

### Immunofluorescence staining

To conduct immunofluorescence staining, brain sections were rinsed in 0.2% PBST (PBS + 0.2% Triton-X 100) and incubated in blocking solution [10% normal goat serum (Vector Laboratories, S-1000-20, Burlingame, CA) in PBST (PBS + 0.2% Triton-X 100)] for 2 h at room temperature. Next, the tissue sections were incubated with primary antibodies at 4°C for 24–72 h, washed with PBST, and incubated with fluorescent goat anti-rabbit, anti-mouse or anti-chicken secondary antibodies conjugated with Alexa Fluor 488 or 555 for 2 h at room temperature. Finally, the brain sections were washed with PBST, PBST/DAPI, and PBS and mounted on glass slides with a mounting solution containing DAPI (Vector Laboratories, H-1200-10, Burlingame, CA). Images of the immunostained tissue were obtained by fluorescence microscopy (DMi8, Leica Microsystems) and quantified using ImageJ software (US National Institutes of Health, Bethesda, MD, USA). The primary and secondary antibodies are listed in **Table 2**.

### ELISA for IL-6 and IL-1β

Cortex and hippocampal tissues from mice treated with lomerizine were lysed in RIPA buffer (Merck Millipore, Billerica, MA, USA) containing 1% protease and phosphatase inhibitor cocktail (Thermo Scientific, Waltham, MA, USA) and incubated for 1 h on ice. The brain lysates were centrifuged at 12000 rpm for 20 min at 4°C, and the supernatant was collected. IL-6 and IL-1β protein levels (100 µg of protein) were measured using IL-6 and IL-1β ELISA kits (IL-1β ELISA kit: 88-7013-88, Invitrogen, Waltham, Massachusetts, USA, IL-6, ELISA kit: 88-7064-88, Invitrogen, Waltham, Massachusetts, USA) according to the manufacturer’s instructions. For ELISA analysis of treated BV2 microglial cells, the culture medium was collected, and IL-1β protein levels in 100 µl of culture medium were measured using an IL-1β ELISA kit (88-7013-88, Invitrogen, Waltham, Massachusetts, USA) according to the manufacturer’s instructions.

### Western blot

To investigate the effects of lomerizine pretreatment on LPS-induced NLRP3 or p-GSK3α/β levels *in vitro*, BV2 microglial cells were treated with lomerizine (10 µM) or vehicle (1% DMSO) for 30 min (NLRP3) or 5.5 h (p-GSK3α/β) followed by LPS (200 ng/ml) or PBS for 30 min (p-GSK3α/β) or 5.5 h (NLRP3). BV2 microglial cells were harvested in lysis buffer (50 mM Tris, pH 7.4, 1% Triton X-100, 2 mM CaCl_2_ and 2 mM MgCl_2_) containing protease and phosphatase inhibitors and centrifuged at 12000 rpm for 15 min. Protein assay reagents (Bio-Rad Laboratories, Hercules, CA, USA) were used to quantify the protein concentration in the supernatant. For western blot analysis, 20 µg of protein sample was boiled at 100°C for 5 min, loaded onto an 8% SDS-PAGE gel, and separated by electrophoresis. The proteins in the gel were electrotransferred to a PVDF membrane (Millipore, Bedford, MA, USA), which was blocked in 5% skim milk and incubated with the primary antibody at 4°C overnight. The next day, the membrane was incubated with HRP-conjugated goat anti-mouse IgG or HRP-conjugated goat anti-rabbit IgG (1:10000, Enzo Life Sciences, Farmingdale, NY, USA), and proteins were detected by adding ECL Western Blotting Detection solution (GE Healthcare, Chicago, IL, USA). Images were acquired and analyzed by Fusion Capt Advance software (Vilber Lourmat).

### Statistical analysis

GraphPad Prism 8 software (GraphPad Software, San Diego, CA, USA) was used for graph generation and statistical analysis. For comparison between two groups, Student’s t test or paired t-test was used. For multiple comparisons, One-way analysis of variance (ANOVA) with Tukey’s, Newman-Keuls or Sidak’s *post hoc* analysis was used. The results are presented as the mean ± SEM (*p < 0.05, **p < 0.01, ***p < 0.001). Detailed statistical analysis information is provided in **Supplementary Table 1**.

## Results

### Pretreatment with lomerizine downregulates LPS-evoked proinflammatory cytokine and NLRP3 mRNA levels in BV2 microglial cells

Before performing *in vitro* experiments, the cytotoxicity of lomerizine in BV2 microglial cells was assessed using the MTT assay. Cell viability was measured after treating BV2 microglial cells with vehicle (1% DMSO) or lomerizine (0.1, 1, 5, 10, or 20 µM) for 24 h. Even at the highest concentration (20 µM), lomerizine had no cytotoxic effects on BV2 microglial cells compared with vehicle (**Figures 1A, B**).

Next, to determine whether lomerizine pretreatment affects LPS-induced proinflammatory cytokine expression, BV2 microglial cells were treated with lomerizine (10 µM) or vehicle (1% DMSO) for 30 min followed by LPS (200 ng/ml) or PBS for 5.5 h. Real-time PCR showed that lomerizine pretreatment significantly reduced the LPS-mediated upregulation of IL-1β, pro-IL-1β, and IL-6 mRNA levels but not COX-2 and TNF-α mRNA levels (**Figures 1C–G**). These data suggest that lomerizine pretreatment reduces LPS-stimulated proinflammatory responses *in vitro*.

With respect to the underlying molecular mechanisms of this effect, real-time PCR showed that lomerizine pretreatment significantly diminished the LPS-induced increase in NLRP3 mRNA levels but had no effect on LPS-induced CDK6 and SOD2 mRNA levels (**Figures 1H–J**). These data indicate that lomerizine pretreatment reduces NLRP3 expression to decrease LPS-mediated proinflammatory cytokine release in microglia.

### Pretreatment with lomerizine suppresses the LPS-induced expression of Iba-1 and GFAP and the proliferation of microglia and astrocytes in wild-type mice

**Figure 2:**
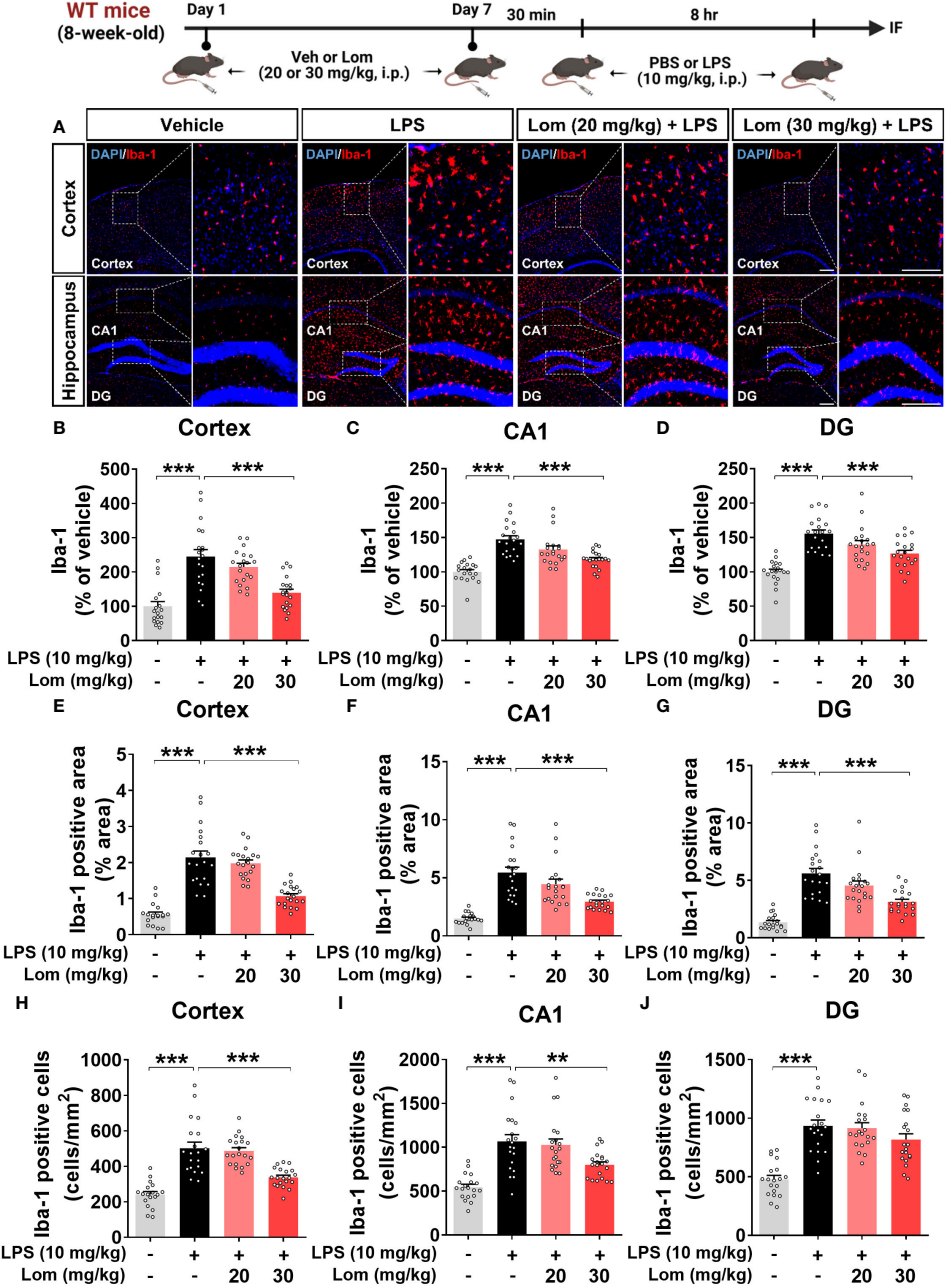
Pretreatment with lomerizine significantly diminishes LPS-mediated Iba-1 fluorescence intensity, Iba-1-positive area, and the number of Iba-1-positive cells in wild-type mice. **(A)** Immunofluorescence staining of Iba-1 in brain tissues of wild-type mice injected with vehicle (2% DMSO, i.p.) or lomerizine (20 or 30 mg/kg, i.p.) daily for 7 days followed by LPS (10 mg/kg, i.p.) or PBS on day 7. **(B–J)** Quantification of data from A (n = 18–20 brain slices from 5 mice/group). **p < 0.01; ***p < 0.001. Scale bar = 200 µm.

Since lomerizine affected LPS-evoked proinflammatory responses *in vitro*, we investigated the impact of lomerizine pretreatment on LPS-induced microgliosis *in vivo*. Wild-type mice were injected with lomerizine (20 or 30 mg/kg, i.p.) or vehicle (2% DMSO, i.p.) daily for 7 days. Thirty minutes after the lomerizine or vehicle injection on day 7, the mice were injected with LPS (10 mg/kg, i.p.) or PBS (i.p.). Eight hours after the LPS injection, the mice were sacrificed, and the expression of Iba-1 and GFAP in brain tissues was examined. We found that LPS treatment significantly increased Iba-1 fluorescence intensity, Iba-1-positive area, and the number of Iba-1-positive cells in both the cortex and hippocampus (**Figures 2A–J**). In addition, pretreatment with 30 mg/kg lomerizine significantly attenuated the LPS-induced increases in Iba-1 fluorescence intensity and the Iba-1-positive area in the cortex and hippocampus; however, lomerizine pretreatment significantly reduced the number of Iba-1-positive cells only in the cortex and the hippocampal CA1 region and not in the hippocampal DG region (**Figures 2A–J**). By contrast, pretreatment with 20 mg/kg lomerizine had no effect on the LPS-mediated increases in Iba-1 fluorescence intensity, Iba-1-positive area, and number of Iba-1-positive cells (**Figures 2A–J**).

**Figure 3:**
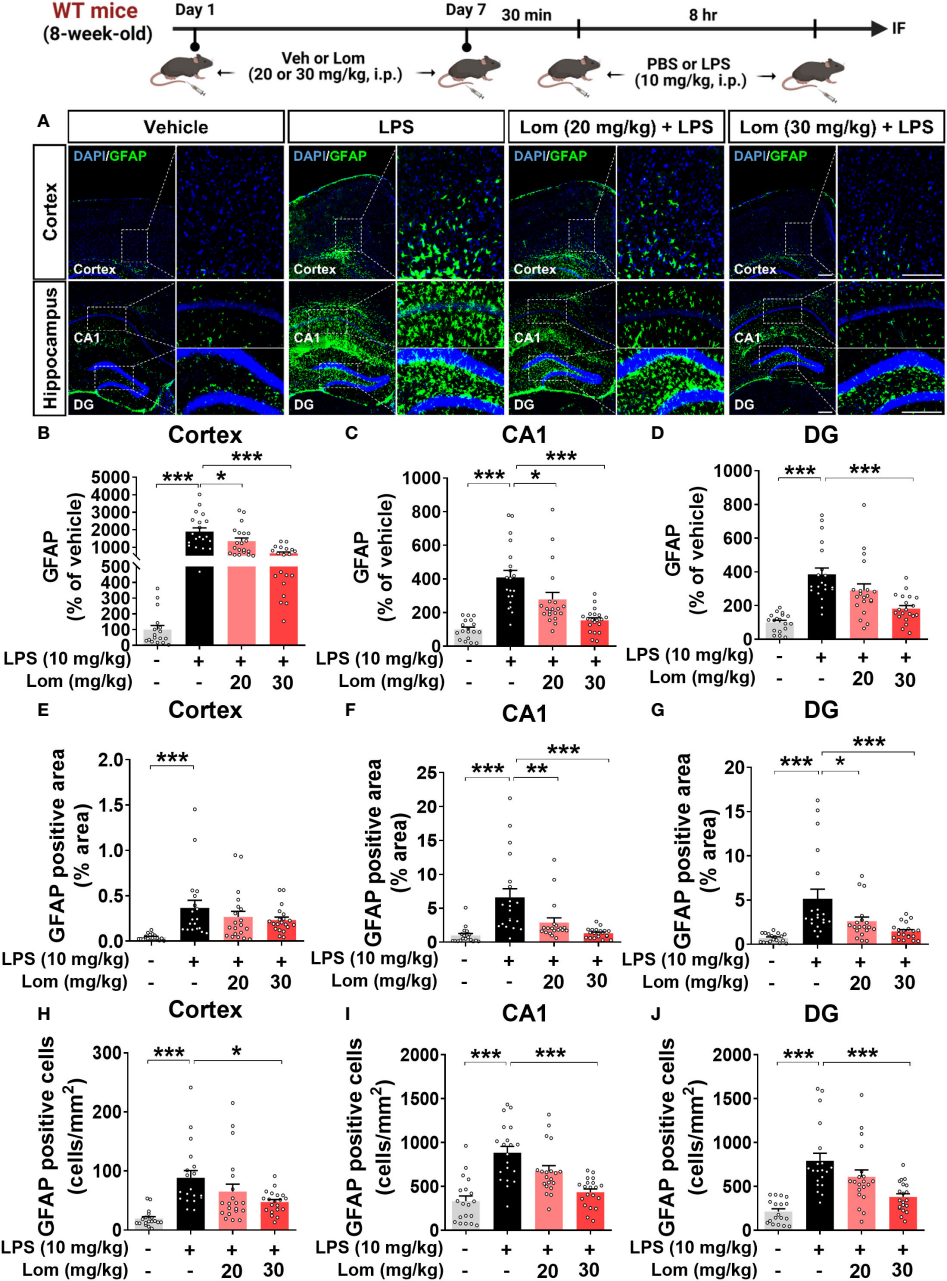
Lomerizine pretreatment significantly decreases LPS-induced GFAP fluorescence intensity, GFAP-positive area, and the number of GFAP-positive cells in wild-type mice. **(A)** Immunofluorescence staining of GFAP in brain tissues of wild-type mice injected with vehicle (2% DMSO, i.p.) or lomerizine (20 or 30 mg/kg, i.p.) daily for 7 days followed by LPS (10 mg/kg, i.p.) or PBS on day 7. **(B–J)** Quantification of data from A (n = 17–20 brain slices from 5 mice/group). *p < 0.05, **p < 0.01, ***p < 0.001. Scale bar = 200 µm.

We then examined whether lomerizine pretreatment modulates LPS-evoked astrogliosis *in vivo*. Pretreatment with 30 mg/kg lomerizine significantly diminished the LPS-induced increases in GFAP fluorescence intensity in the cortex and hippocampus (**Figures 3A–D**). In addition, both doses of lomerizine pretreatment significantly reduced the LPS-mediated increase in the GFAP-positive area in the hippocampus but not in the cortex (**Figures 3E–G**). Moreover, pretreatment with 30 mg/kg lomerizine significantly downregulated LPS-induced the number of GFAP-positive cells in the cortex and hippocampus (**Figures 3H–J**). Thus, lomerizine pretreatment ameliorated LPS-induced microglial and astrocyte activation in wild-type mice.

### Pretreatment with lomerizine diminishes LPS-induced proinflammatory cytokine levels in wild-type mice

To determine whether lomerizine itself modulates proinflammatory responses *in vivo*, wild-type mice were injected with lomerizine (30 mg/kg, i.p.) or vehicle (2% DMSO) daily for 7 days, and the dissected cortex and hippocampal tissues were analyzed by ELISA. We found that lomerizine treatment alone did not alter the expression of the proinflammatory cytokines IL-6 and IL-1β, suggesting that lomerizine itself does not alter the inflammatory response in the CNS *in vivo* (**Supplementary Figure 1**).

**Figure 4:**
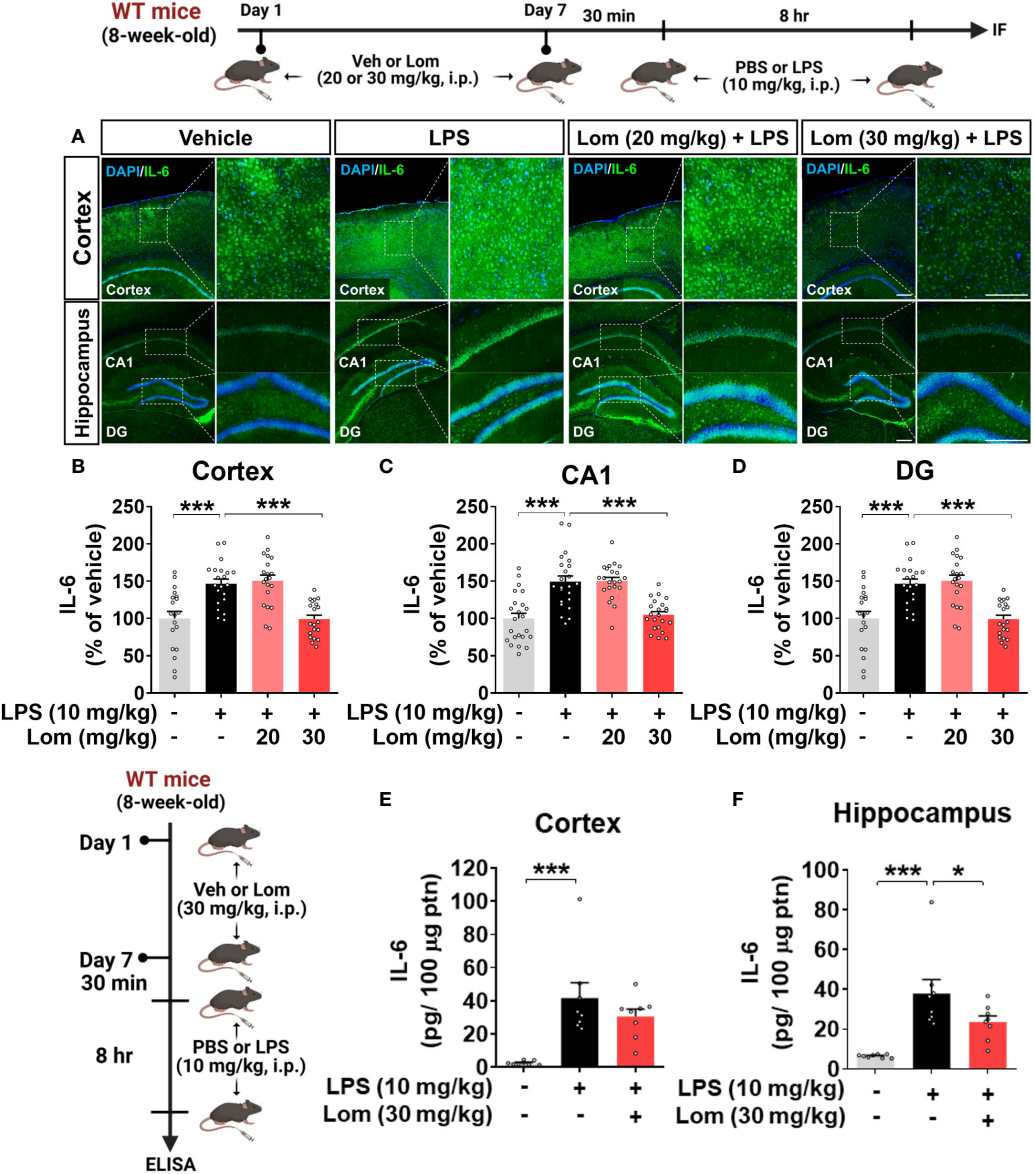
Pretreatment with lomerizine significantly reduces LPS-induced proinflammatory cytokine IL-6 levels in wild-type mice. **(A)** Wild-type mice were injected with vehicle (2% DMSO, i.p.) or lomerizine (20 or 30 mg/kg, i.p.) daily for 7 days followed by LPS (10 mg/kg, i.p.) or PBS on day 7. Immunofluorescence staining of IL-6 in brain tissues. **(B–D)** Quantification of data from A (n = 19–23 brain slices from 5 mice/group). **(E, F)** ELISA analysis of IL-6 levels in the cortex and hippocampus (n = 8/group). *p < 0.05, ***p < 0.001. Scale bar = 200 µm.

We then investigated whether lomerizine pretreatment modulates LPS-mediated proinflammatory cytokine levels *in vivo*. Wild-type mice were injected with lomerizine (20 or 30 mg/kg, i.p.) or vehicle (2% DMSO, i.p.) daily for 7 days. Thirty minutes after the injection of lomerizine or vehicle on day 7, the mice were injected with LPS (10 mg/kg, i.p.) or PBS. Eight hours after the LPS or PBS injection, the mice were sacrificed, and immunofluorescence staining of brain tissues was performed with an anti-IL-6 antibody. Wild-type mice pretreated with 30 mg/kg lomerizine exhibited a significant decrease in the LPS-induced expression of the proinflammatory cytokine IL-6 in the cortex and hippocampus (**Figures 4A–D**). By contrast, pretreatment with 20 mg/kg lomerizine did not attenuate the LPS-induced upregulation of IL-6 in wild-type mice (**Figures 4A–D**).

To further confirm these findings, IL-6 protein levels in the cortex and hippocampus of mice pretreated with lomerizine as described above were assessed by ELISA. Pretreatment with 30 mg/kg lomerizine significantly reduced LPS-evoked IL-6 levels in the hippocampus but not in the cortex of wild-type mice (**Figures 4E, F**). These data indicate that pretreatment with 30 mg/kg lomerizine modulates LPS-evoked proinflammatory cytokine IL-6 levels *in vivo*.

### Pretreatment with lomerizine decreases NLRP3 expression in LPS-treated wild-type mice

**Figure 5:**
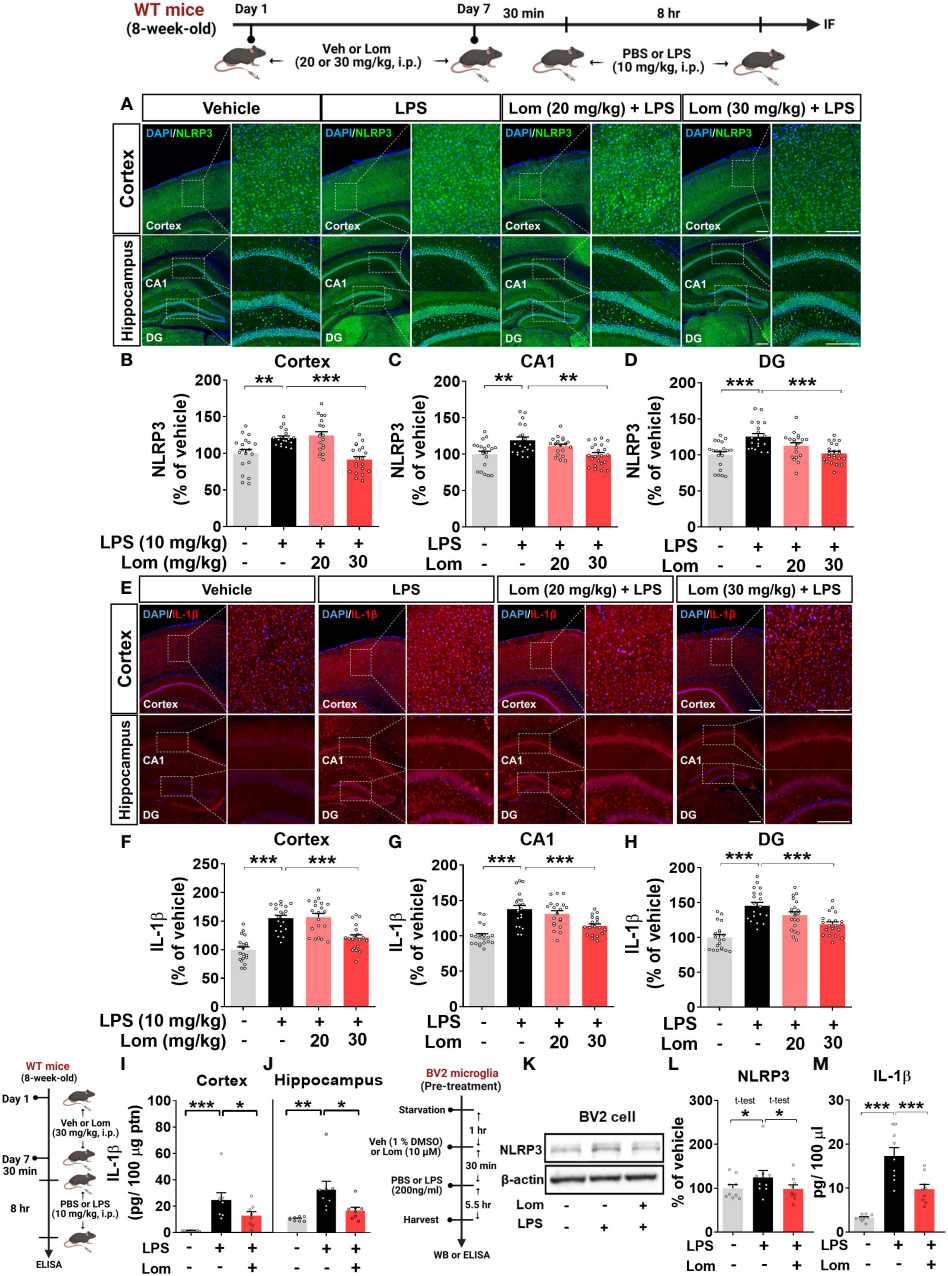
Lomerizine pretreatment significantly reduces LPS-mediated NLRP3 and IL-1β expression *in vitro* and *in vivo*. **(A–J)** Wild-type mice were injected with vehicle (2% DMSO, i.p.) or lomerizine (20 or 30 mg/kg, i.p.) daily for 7 days followed by LPS (10 mg/kg, i.p.) or PBS on day 7. **(A)** Immunofluorescence staining of NLRP3 in brain tissues. **(B–D)** Quantification of data from A (n = 19–22 brain slices from 5 mice/group). **(E)** Immunofluorescence staining of IL-1β in brain tissues. **(F–H)** Quantification of data from E (n = 20 brain slices from 5 mice/group). **(I, J)** ELISA analysis of IL-1β levels in the cortex and hippocampus (n = 8/group). **(K–M)** BV2 microglial cells were treated with vehicle (1% DMSO) or lomerizine (10 µM) for 30 min followed by LPS (200 ng/ml) or PBS for 5.5 h (n = 9/group). **(K)** Representative immunoblots image of NLPR3 and β-actin. **(L)** Western blot analysis of NLRP3 protein levels. **(M)** ELISA analysis of IL-1β levels. *p < 0.05, **p < 0.01, ***p < 0.001. Scale bar = 200 µm.

Since lomerizine inhibited NLRP3 upregulation in LPS-treated BV2 microglial cells, we explored the effects of lomerizine on LPS-primed NLRP3 expression *in vivo*. For this experiment, wild-type mice were treated with lomerizine (20 or 30 mg/kg, i.p.) or vehicle (2% DMSO, i.p.) daily for 7 days; on day 7, the mice were injected with LPS (10 mg/kg, i.p.) or PBS 30 min after the lomerizine or vehicle injection. Eight hours later, the mice were sacrificed, and the expression of NLRP3 in the cortex and hippocampus was assessed by immunofluorescence staining. Pretreatment with 30 mg/kg lomerizine significantly reduced LPS-evoked NLRP3 fluorescence intensity in the cortex and hippocampal CA1 and DG regions in wild-type mice (**Figures 5A–D**). However, 20 mg/kg lomerizine pretreatment had no impact on LPS-primed NLRP3 expression in the brain (**Figures 5A–D**).

We then examined the effects of lomerizine pretreatment on LPS-mediated IL-1β levels *in vivo*. Pretreatment with 30 mg/kg but not 20 mg/kg lomerizine significantly decreased LPS-evoked IL-1β levels in the cortex and hippocampus in wild-type mice (**Figures 5E–H**). To verify these findings, IL-1β levels in the cortex and hippocampus were assessed by ELISA. We found that 30 mg/kg lomerizine pretreatment significantly diminished LPS-evoked IL-1β levels (**Figures 5I–J**). These results indicate that lomerizine pretreatment can attenuate LPS-primed NLRP3 and IL-1β expression *in vivo*.

To validate the *in vivo* findings, the effects of lomerizine on LPS-mediated NLRP3 and IL-1β levels were evaluated *in vitro*. After pretreatment with lomerizine (10 µM) or vehicle (1% DMSO) for 30 min, BV2 microglial cells were treated with LPS (200 ng/ml) or PBS for 5.5 h, and western blotting and ELISA were conducted. Lomerizine pretreatment significantly attenuated LPS-mediated NLRP3 and IL-1β levels in BV2 microglial cells (**Figures 5K–M**), indicating that lomerizine downregulates LPS-primed NLRP3 and IL-1β expression both *in vivo* and *in vitro*.

### Lomerizine pretreatment downregulates proinflammatory cytokine levels induced by intracerebroventricular LPS injection in wild-type mice

To determine whether lomerizine can modulate neuroinflammation induced by direct intracerebroventricular (i.c.v.) injection of LPS in the brain, wild-type mice were first pretreated with lomerizine (30 mg/kg, i.p.) or vehicle (2% DMSO, i.p.) by daily injection for 14 consecutive days. On day 14, saline or LPS (3 µg in 1 µl of saline/lateral ventricle) was administered *via* bilateral i.c.v. injection 2 h after the lomerizine or vehicle injection. Four hours after the LPS or saline injection, the mice were sacrificed, the cortex and hippocampus were dissected, and ELISA was performed. We found that 14 days of daily lomerizine pretreatment significantly decreased i.c.v.-administered LPS-evoked hippocampal IL-1β and cortical/hippocampal IL-6 protein levels, whereas cortical IL-1β protein levels were not altered (**Supplementary Figures 2A–D**). These data indicate that lomerizine can cross the BBB and effectively modulate LPS-mediated proinflammatory responses in the brain in wild-type mice.

### Posttreatment with lomerizine diminishes LPS-mediated proinflammatory cytokine levels in BV2 microglial cells and wild-type mice

**Figure 6:**
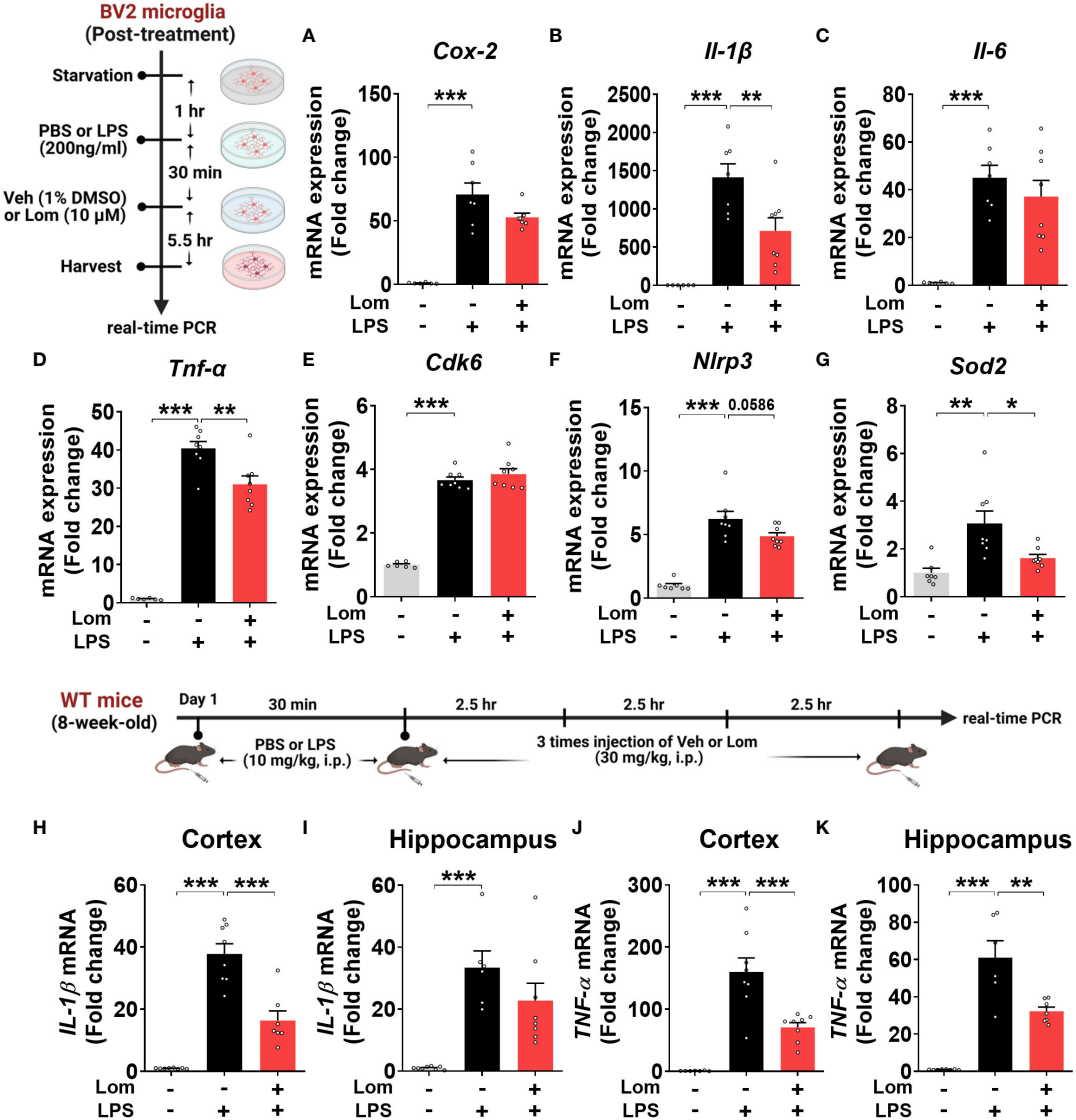
Posttreatment with lomerizine diminishes LPS-evoked proinflammatory cytokine levels in BV2 microglial cells and wild-type mice. **(A–G)** BV2 microglial cells were treated with LPS (200 ng/ml) or PBS for 30 min followed by vehicle (1% DMSO) or lomerizine (10 µM) for 5.5 h (n = 6–8/group). **(A–D)** Real-time PCR analysis of proinflammatory cytokine mRNA levels. **(E–G)** Real-time PCR analysis of CDK6, NLRP3, and SOD2 mRNA levels. **(H–K)** Real-time PCR of IL-1β and TNF-α mRNA levels in the cortex and hippocampus in wild-type mice injected with LPS (10 mg/kg, i.p.) or PBS followed by vehicle (2% DMSO, i.p.) or lomerizine (30 mg/kg, i.p.) (n = 6–8/group). *p < 0.05, **p < 0.01, ***p < 0.001.

To determine whether lomerizine posttreatment could affect LPS-induced proinflammatory responses *in vitro*, BV2 microglial cells were treated with LPS (200 ng/ml) or PBS for 30 min and posttreated with lomerizine (10 µM) or vehicle (1% DMSO) for 5.5 h. Subsequent real-time PCR showed that lomerizine posttreatment significantly suppressed the LPS-mediated upregulation of IL-1β and TNF-α mRNA levels but not COX-2 and IL-6 mRNA levels (**Figures 6A–D**). These data suggest that lomerizine posttreatment attenuates LPS-evoked proinflammatory responses *in vitro*.

Next, we assessed the effects of lomerizine posttreatment on molecular targets associated with LPS-induced neuroinflammatory responses in BV2 microglial cells. Real-time PCR revealed that lomerizine posttreatment significantly reduced the LPS-induced increase in SOD2 mRNA levels but had no effect on NLRP3 and CDK6 mRNA levels (**Figures 6E–G**). These data indicate that lomerizine posttreatment reduces SOD2 expression to reduce LPS-mediated proinflammatory cytokine release *in vitro*.

To examine the effects of lomerizine posttreatment on LPS-stimulated proinflammatory responses *in vivo*, wild-type mice were injected with LPS (10 mg/kg, i.p.) or PBS; 30 min later, the mice were injected with lomerizine (30 mg/kg, i.p.) or vehicle (2% DMSO) three times at 2.5-h intervals. The mice were sacrificed 2.5 h after the final lomerizine or vehicle injection, and the cortex and hippocampus were dissected. Real-time PCR analysis showed that lomerizine posttreatment significantly reduced LPS-stimulated IL-1β in the cortex and TNF-α mRNA levels in both cortex and hippocampus in wild-type mice (**Figures 6H–K**), suggesting that lomerizine posttreatment also modulates LPS-evoked proinflammatory responses in wild-type mice with different mechanisms compared with its pretreatment.

### Pretreatment with lomerizine suppresses LPS-induced tau hyperphosphorylation in wild-type mice

**Figure 7:**
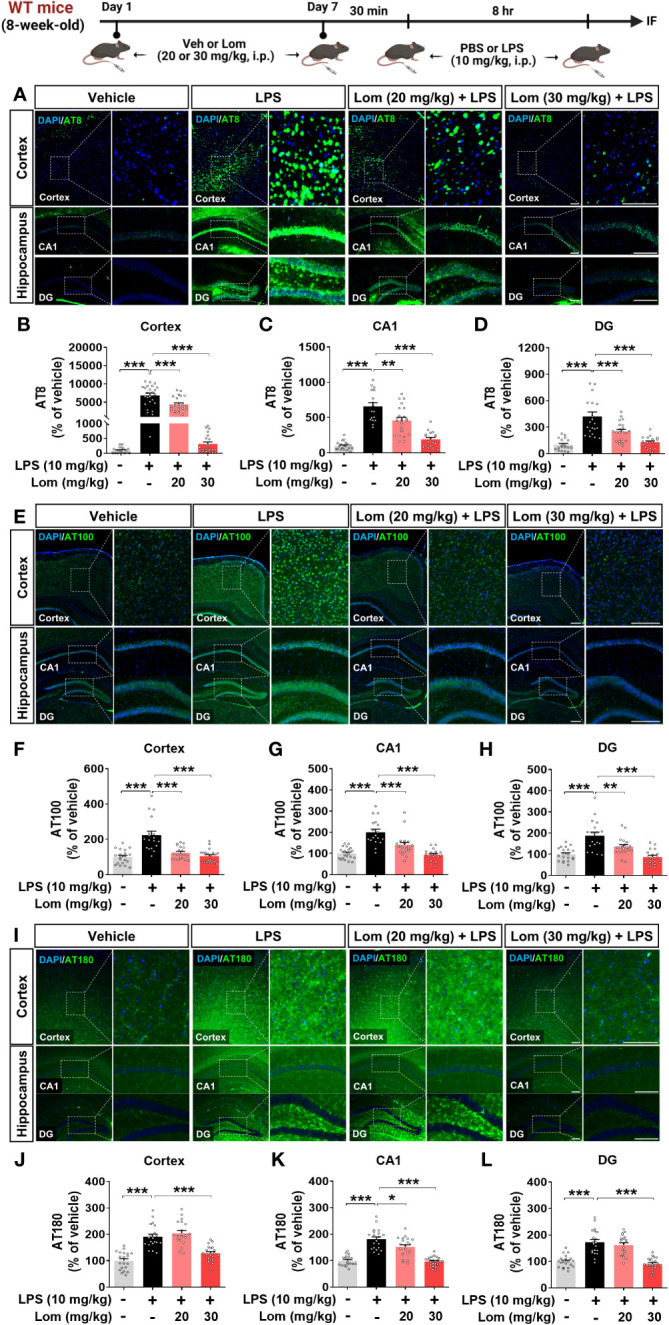
Lomerizine pretreatment significantly decreases LPS-mediated tau hyperphosphorylation in wild-type mice. **(A–L)** Wild-type mice were injected with vehicle (2% DMSO, i.p.) or lomerizine (20 or 30 mg/kg, i.p.) daily for 7 days followed by LPS (10 mg/kg, i.p.) or PBS on day 7. **(A, E, I)** Immunofluorescence staining of brain tissues with anti-AT8, anti-AT100, and anti-AT180 antibodies. **(B–D)** Quantification of data from A (n = 17–26 brain slices from 5 mice/group). **(F–H)** Quantification of data from E (n = 18–20 brain slices from 5 mice/group). **(J–L)** Quantification of data from I (n = 19–24 brain slices from 5 mice/group). *p < 0.05, **p < 0.01, ***p < 0.001. **(A, I)** Scale bar = 100 µm, **(E)** Scale bar = 200 µm.

Aberrant tau hyperphosphorylation induces tau-dependent pathology, which is associated with cognitive function decline and neuroinflammation (14). Interestingly, several recent studies have demonstrated that LPS not only exacerbates tauopathy in an AD mouse model but also affects tau hyperphosphorylation in wild-type mice (15, 16). To examine the effects of lomerizine pretreatment on LPS-induced tau hyperphosphorylation *in vivo*, wild-type mice were injected with lomerizine (20 or 30 mg/kg, i.p.) or vehicle (2% DMSO, i.p.) daily for 7 days; on day 7, LPS (10 mg/kg, i.p.) or PBS was injected 30 min after the final lomerizine or vehicle injection. Eight hours after the LPS or PBS injection, the mice were sacrificed and perfused for immunofluorescence staining with anti-AT8 (Ser202/Thr205), anti-AT100 (Thr212/Ser214), and anti-AT180 (Thr231) antibodies. Importantly, LPS treatment significantly increased tau hyperphosphorylation in wild-type mice compared with vehicle treatment (**Figures 7A–L**). In addition, pretreatment with 20 or 30 mg/kg lomerizine significantly reduced LPS-induced tau hyperphosphorylation at Ser202/Thr205 (**Figures 7A–D**) and Thr212/Ser214 in the cortex and hippocampus of wild-type mice (**Figures 7E–H**). Moreover, 30 mg/kg lomerizine pretreatment also protected against tau hyperphosphorylation at Thr231 in the cortex and hippocampus, whereas 20 mg/kg lomerizine pretreatment reduced tau hyperphosphorylation at Thr231 only in the hippocampal CA1 region (**Figures 7I–L**). These findings suggest that lomerizine pretreatment mitigates LPS-induced tau hyperphosphorylation *in vivo*.

### Pretreatment with lomerizine inhibits the LPS-induced activation of the tau kinases GSK3α/β and DYRK1A *in vivo*

**Figure 8:**
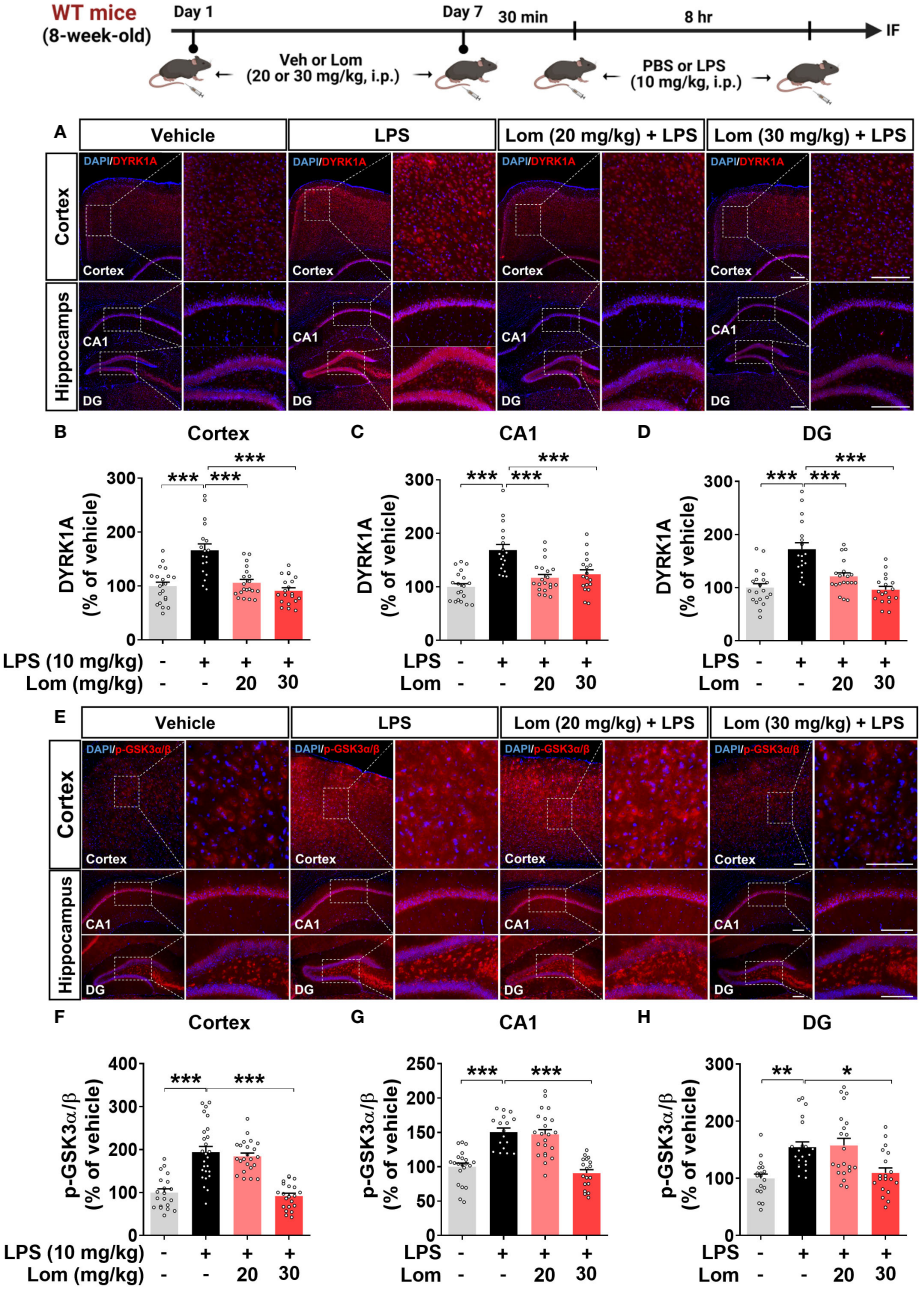
Lomerizine pretreatment significantly reduces LPS-mediated tau kinase DYRK1A and GSK3α/β activation in the brain of wild-type mice. **(A, E)** Immunofluorescence staining of DYRK1A and GSK3α/β in brain tissues from wild-type mice injected with vehicle (2% DMSO, i.p.) or lomerizine (20 or 30 mg/kg, i.p.) daily for 7 days followed by LPS (10 mg/kg, i.p.) or PBS. **(B–D)** Quantification of data from A (n = 18–20 brain slices from 5 mice/group). **(F–H)** Quantification of data from E (n = 17–26 brain slices from 5 mice/group). *p < 0.05, **p < 0.01, ***p < 0.001. **(A)** Scale bar = 200 µm, **(E)** Scale bar = 100 µm.

We next explored the potential molecular mechanisms underlying the attenuation of LPS-induced tau phosphorylation by lomerizine *in vivo*. Wild-type mice were injected with lomerizine (20 or 30 mg/kg, i.p.) or vehicle (2% DMSO, i.p.) daily for 7 days and, on day 7, injected with LPS (10 mg/kg, i.p.) or PBS 30 min after the lomerizine or vehicle injection. Eight hours later, the mice were sacrificed, and the expression of the tau kinases DYRK1A and p-GSK3α/β (as an antibody recognizing both p-GSK3β and p-GSK3α was used) in brain tissues was quantified. Both 20 and 30 mg/kg lomerizine pretreatment significantly downregulated LPS-induced DYRK1A expression in wild-type mice (**Figures 8A–D**). In addition, 30 mg/kg lomerizine pretreatment significantly suppressed LPS-mediated GSK3α/β phosphorylation in the cortex and hippocampus (**Figures 8E–H**). These results indicate that lomerizine pretreatment inhibits LPS-induced tau hyperphosphorylation in wild-type mice by downregulating the tau kinases DYRK1A and GSK3α/β.

### Lomerizine reduces tau hyperphosphorylation in human AD neurons

**Figure 9:**
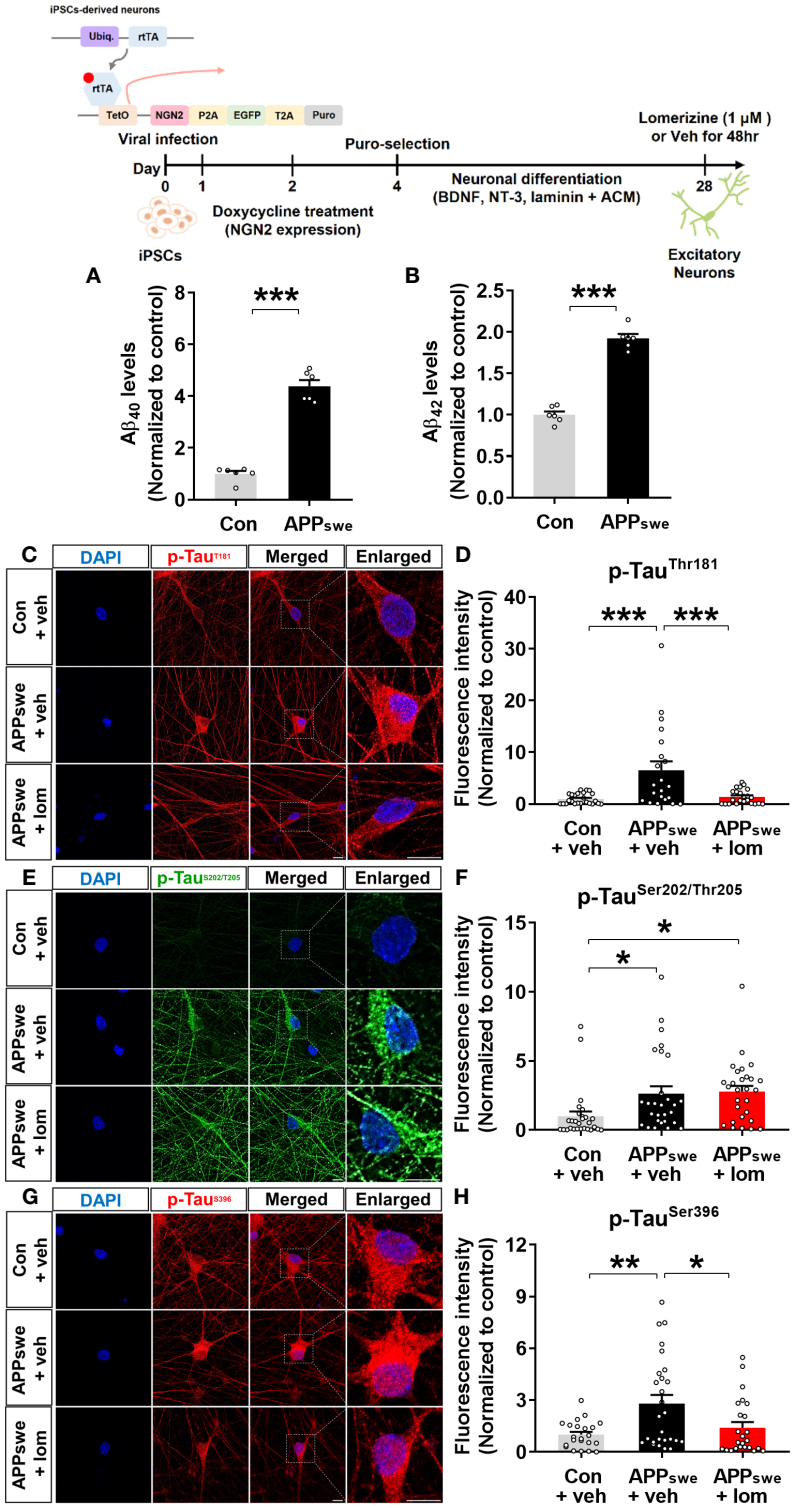
Lomerizine significantly downregulates tau hyperphosphorylation in AD neurons derived from human iPSCs. **(A, B)** Aβ_40_ **(A)** and Aβ_42_ **(B)** levels in isogenic control and AD neurons (n = 6/group**). (C, E, G)** Isogenic control or AD neurons were treated with vehicle (1% DMSO) or lomerizine (1 μM) for 48 h, and immunofluorescence staining was performed with anti-p-Tau^Thr181^, anti-p-Tau^Ser202/Thr205^, and anti-p-Tau^Ser396^ antibodies. **(D)** Quantification of data from C (n = 21–24/group). **(F)** Quantification of data from E (n = 28/group). **(H)** Quantification of data from G (n = 22–27/group). *p < 0.05, **p < 0.01, ***p < 0.001. Scale bar = 10 µm.

To validate the effects of lomerizine on tau hyperphosphorylation in the human context, we utilized human iPSCs to generate a model of neurodegeneration. Specifically, we generated AD neurons by using CRISPR/Cas9 genome editing to insert the familial AD-associated APPswe mutation (APP KM670/671NL) into iPSCs and differentiating the iPSCs into excitatory neurons. Measurement of Aβ levels in APPswe and isogenic control neurons by ELISA showed that Aβ_40_ and Aβ_42_ levels were significantly higher in APPswe neurons than in isogenic control neurons (**Figures 9A, B**).

Next, the effects of lomerizine on tau phosphorylation were evaluated in isogenic control and APPswe neurons treated with vehicle (1% DMSO) or lomerizine (1 μM) for 48 h. immunocytochemistry with anti-p-tau antibodies demonstrated that tau phosphorylation at Thr181, Ser202/Thr205 (AT8), and Ser396 was significantly higher in APPswe neurons than in isogenic control neurons beginning at 4 weeks of differentiation (**Figures 9C–H**). Moreover, treatment with lomerizine (1 μM) significantly reduced tau phosphorylation at Thr181 (**Figures 9C, D**) and Ser396 (**Figures 9G, H**) but not at Ser202/Thr205 in APPswe neurons compared with isogenic control neurons (**Figures 9E, F**). These observations suggest that lomerizine may have beneficial effects on tauopathy in human neurons and is a potential drug for tauopathy-associated diseases.

## Discussion

Acute neuroinflammation is a defensive reaction regulated by innate immunity and protective autoimmunity to protect the nervous system against infectious insults (17). However, chronic neuroinflammation is associated with neuronal death and thus contributes to the development of neurodegenerative diseases, including AD, Parkinson’s disease (PD), and epilepsy (17, 18). Microglia are innate immune cells of the CNS that defend the brain from harmful pathogens and cellular detritus and contribute to CNS homeostasis (19). Microglial activation is induced by various pathological states in the CNS, including injury, ischemia, neuronal damage and stimuli such as LPS (20, 21). Chronic or excessive microglial activation has been implicated in the pathoprogression of neurodegenerative diseases (22).

Several lines of evidence suggest a correlation between intracellular Ca^2+^ levels and proinflammatory responses. For instance, increased intracellular Ca^2+^ levels enhance the expression of the proinflammatory cytokines IL-1β and TNF-α (23). *In vitro* studies have shown that increased intracellular Ca^2+^ levels induce microglial activation and neuronal death or injury by eliciting the release of inflammatory molecules (24). Moreover, LPS increases the levels of proinflammatory cytokines such as IL-1β, TNF-α, COX-2, and iNOS and intracellular Ca^2+^ concentrations in microglia. Accordingly, beneficial effects of calcium channel blockers on neuroinflammation have been reported. Nicardipine attenuates the upregulation of proinflammatory cytokines such as COX-2, IL-6, and IL-1β and NO production induced by co-treatment with LPS and IFN-γ in BV2 microglial cells and the mouse brain (25). Zn^2+^, a potential calcium channel blocker in neurons, and Zn^2+^-containing compounds inhibit COX-2 expression and ROS production in LPS-treated BV2 microglial cells. (26, 27). In addition, pretreatment with cilnidipine, an N/L-type calcium channel blocker, suppresses the induction of IL-1β expression by co-treatment with LPS and ATP in primary microglia (28). Furthermore, the calcium channel blockers verapamil and diltiazem significantly downregulate quinolinic acid-induced TNF-α and IL-6 levels in a rat model of Huntington’s disease (29). This study is the first to examine the effects of the L- and T-type voltage-gated calcium channel blocker lomerizine on LPS-stimulated proinflammatory cytokine levels *in vivo* and *in vitro*.

We found that pre- and posttreatment with lomerizine significantly reduced LPS-induced proinflammatory cytokine levels in BV2 microglial cells (**Figures 1**, **6**). Interestingly, lomerizine pretreatment downregulated LPS-evoked IL-1β mRNA/protein and IL-6 mRNA levels, whereas lomerizine posttreatment suppressed LPS-mediated IL-1β and TNF-α mRNA levels (**Figures 1**, **6**). How do pre- and posttreatment with lomerizine differentially regulate LPS-evoked proinflammatory responses *in vitro*? It is likely that the molecular targets modulated by pre- and posttreatment differ; for example, pretreatment reduced NLRP3 expression (**Figure 1**), whereas its posttreatment reduced SOD2 expression induced by LPS (**Figure 6**). Future knock-down and knock-out experiments will demonstrate whether lomerizine pre- or posttreatment regulates LPS-evoked proinflammatory responses in an NLRP3- or SOD2-dependent manner.

Furthermore, we found that although lomerizine itself did not affect neuroinflammation *in vivo* (**Supplementary Figure 1**), 30 mg/kg lomerizine pretreatment (i.p.) significantly downregulated cortical IL-6 and IL-1β expression in LPS (i.p. and i.c.v.)-injected wild-type mice, suggesting that lomerizine can cross the BBB and effectively modulate LPS-mediated proinflammatory responses in the brain (**Figures 4**, **5**, and **Supplementary Figure 2**). Overall, the current findings indicate that lomerizine attenuates the LPS-induced upregulation of proinflammatory cytokines *in vivo* and *in vitro* and thus may have preventative and therapeutic effects on neuroinflammation. In future work, we will investigate whether lomerizine modulates anti-inflammatory responses *in vitro* and *in vivo*.

In the CNS, microglia and astrocytes play crucial roles in regulating inflammatory responses (30). Acute inflammatory reactions, including the release of cytokines and growth factors, promote recovery after tissue injury (31). However, chronic or unregulated inflammatory responses can cause various neurodegenerative diseases, including AD (31). Activated microglia release proinflammatory cytokines such as TNF-α, IL-1β, and IL-16 and chemokines in response to inflammatory stimuli like LPS (30, 32). Similarly, in many CNS pathologies, proinflammatory cytokines secreted from microglia activate astrocytes, which induces the excessive release of inflammatory factors, including IFN-γ, TNF, and IL-12 (33–35). The deletion of Cav1.2 (calcium channel, voltage-dependent, L type, alpha 1C subunit) attenuates astrogliosis induced by LPS and cuprizone in the brain in mice (36). Overall, previous studies have demonstrated that regulating microglial and astrocyte activity is an important component of treating neuroinflammation and neurodegenerative disorders and that modulating calcium flux is a key factor in the microglial and astrocyte activation response (19, 31, 37–42). The present study is the first to assess the effects of lomerizine on LPS-stimulated microglial and astrocyte activation *in vivo*. We found that lomerizine pretreatment significantly inhibited microglial and astrocyte activation in LPS-treated wild-type mice (**Figures 2**, **3**). Taken together, the literature and our findings suggest that L- and T-type voltage-gated calcium channel blockers can attenuate LPS-stimulated glial activation *in vivo*.

A recent study demonstrated that LPS-induced neuronal loss is reduced in NLRP3^-/-^ mice compared with wild-type mice (43). The NLRP3 inflammasome is a key regulator of inflammatory responses in the CNS and the production of IL-1β (44). The NLRP3 inflammasome is activated by various stimuli, such as pathogen-associated molecular patterns (PAMPs), danger-associated molecular patterns (DAMPs), LPS, and Ca^2+^ signaling pathways (44, 45). Increased extracellular Ca^2+^ can induce NLRP3 inflammasome activation, thereby promoting IL-1β expression; however, this activation is suppressed by the Ca^2+^ blocker 2APB (46–48). A recent study found that nicardipine and nimodipine significantly attenuate NLRP3 inflammasome activation in LPS-stimulated THP-1 cells and Aβ-stimulated microglia, respectively (49). Thus, previous research suggests that calcium channel blockers can modulate LPS/Aβ-mediated proinflammatory responses by reducing NLRP3 inflammasome activation. Our investigation of the impact of lomerizine pretreatment on neuroinflammatory responses revealed that lomerizine pretreatment downregulated LPS-primed NLRP3 and IL-1β expression in BV2 microglial cells and wild-type mice (**Figures 1**, **5**). While lomerizine posttreatment did not significantly alter LPS-induced NLRP3 mRNA expression in BV2 microglial cells (**Figure 6**).

Our findings on the effects of lomerizine pre- and posttreatment on NLRP3 expression give rise to the following question: how do lomerizine (Ca^2+^ channel blocker) pretreatment and posttreatment differentially modulate neuroinflammation-associated molecular targets? Given that cytosolic Ca^2+^ is essential for NLRP3 activation, it is possible that blocking Ca^2+^ channels prior to LPS stimulation (i.e., lomerizine pretreatment) reduces neuroinflammatory responses more effectively than Ca^2+^ channel inhibition after LPS administration (i.e., lomerizine posttreatment). Specifically, increasing calcium mobilization through an influx of extracellular Ca^2+^ *via* calcium channels or the release of intracellular Ca^2+^ from the ER promotes NLRP3 inflammasome assembly and accelerates mitochondrial dysfunction, resulting in NLRP3 activation (50, 51). Therefore, attenuating LPS-primed NLRP3 expression and subsequent proinflammatory response by lomerizine pretreatment can be more efficiently than reversing those alterations by lomerizine posttreatment. Similarly, we previously demonstrated that pretreatment with the L-type Ca^2+^ channel blocker felodipine downregulates the LPS-evoked proinflammatory response in BV2 microglial cells more effectively than posttreatment (52). Future studies require addressing whether lomerizine modulates LPS/ATP-evoked NLRP3 inflammasome assembly (NLRP3/ASC/Caspase-1) and activation *in vitro* and *in vivo*.

Tauopathies, such as the aggregation of insoluble tau due to hyperphosphorylation and misfolding, are hallmarks of AD and induce the formation of neuritic plaques and NFTs (53, 54). During the early phase of AD, hyperphosphorylation of tau and aggregation to form NFTs cause neuronal dysfunction, cognitive deficits, and progression to dementia (55). In humanized tau P301S mutant mice, glial activation is the earliest symptom of neurodegeneration associated with tauopathies (56). Aberrant calcium signaling also induces tau pathology. Ca^2+^ treatment stimulates tau hyperphosphorylation in N2a cells and the brain in wild-type mice (57). Dysregulation of Ca^2+^ homeostasis induces tau phosphorylation and NFT aggregation (58). Furthermore, a recent study found that the calcium channel blocker tetrandrine significantly reduces insoluble phosphorylated tau levels in Thy1-hTau.P301S transgenic mice in a dose-dependent manner (59). Likewise, increased tau protein phosphorylation is significantly downregulated by the calcium channel blocker verapamil in 5xFAD mice (60). Interestingly, several recent studies have shown that LPS affects tau phosphorylation *in vitro* and *in vivo*. For example, neurons co-cultured with LPS-stimulated microglia display increased tau hyperphosphorylation and synaptic deficits (61). In wild-type mice, LPS differentially regulates tau hyperphosphorylation in an injection period- or dose-dependent manner. For example, tau phosphorylation at Ser202/Thr205 and Ser422 significantly increases 20 min after the injection of 100 μg/kg LPS (i.p.) but returns to basal levels at 180 min after LPS injection (16). Another study found that tau phosphorylation at Ser202/Thr205 and Thr231 in the hippocampus in wild-type mice was not altered 24 h after the injection of 1 mg/kg LPS (i.p.) but tended to increase 24 h after injection of a higher dose (10 mg/kg LPS, i.p.) (15). Therefore, in the present study, we wanted to investigate whether the injection of 10 mg/kg LPS (i.p., 8 h), which evokes a sufficient CNS inflammatory response *in vivo*, increases tau phosphorylation in wild-type mice and whether the effects of LPS are modulated by lomerizine pretreatment (30 mg/kg, i.p.). This study is the first to report that lomerizine pretreatment significantly suppresses tau hyperphosphorylation in LPS-treated wild-type mice and AD neurons (**Figures 7**, **9**). In a future study, we will build on these findings by examining the effects of lomerizine on AD pathology (Aβ accumulation as well as tau) in mouse models of AD (e.g., Aβ-overexpressing 5xFAD mice or tau Tg PS19 mice).

Although the tau kinases DYRK1A and GSK3α/β play key roles in the effects of LPS in the CNS and neurodegenerative diseases such as AD, few studies have investigated the effects of calcium channel blockers on tau kinase activity *in vivo* (62). Following our observation that lomerizine treatment significantly attenuated tau hyperphosphorylation, we discovered that lomerizine alleviated LPS-stimulated tau pathology at least partially by reducing the activity/expression of DYRK1A and GSK3α/β (**Figure 8**). It is possible that lomerizine modulates other tau kinases and/or tau-associated molecules to diminish tau phosphorylation, which will be addressed in future studies. Overall, these data indicate that lomerizine regulates the neuroinflammatory response and tau pathology by inhibiting glial activation and the tau kinases DYRK1A and GSK3α/β.

## Conclusion

This study is the first to examine the effects of lomerizine on LPS-stimulated neuroinflammation and tau hyperphosphorylation *in vivo*. We found that lomerizine treatment suppressed LPS-evoked microgliosis/astrogliosis and upregulated proinflammatory cytokine and NLRP3/SOD2 expression *in vitro* and/or *in vivo*. In addition, lomerizine treatment had beneficial effects on tau hyperphosphorylation in LPS-injected wild-type mice and in human AD neurons, potentially through inhibition of the tau kinases GSK3α/β and DYRK1A. These results suggest that lomerizine is a potential therapeutic drug for neuroinflammation and tauopathy-associated diseases.

## Data availability statement

The original contributions presented in the study are included in the article/**Supplementary Material**. Further inquiries can be directed to the corresponding authors.

## Ethics statement

All experimental procedures were approved by the institutional biosafety committee (IBC) and performed in accordance with approved animal protocols of the Korea Brain Research Institute (KBRI, approval no. IACUC-20-00061).

## Author contributions

H-SH and JS conceived and participated in the design of the study. H-SH, JS, J-HP, H-jL, and J-WH wrote the manuscript. J-HP, J-WH, GMJ, YJ, H-jL, and JHC performed experiments and confirmed all data analyses in all figures and the supporting information. GMJ, JHC, and JS generated AD neurons and performed and analyzed related experiments. All authors read and approved the final manuscript. All authors contributed to the article.
