# Supplementary material for: Lomerizine inhibits LPS-mediated neuroinflammation and tau hyperphosphorylation by modulating NLRP3, DYRK1A, and GSK3α/β
Source: Front Immunol. 2023 Jun 26;14:1150940. doi: 10.3389/fimmu.2023.1150940 (PMC10331167; doi:10.3389/fimmu.2023.1150940)
Supplement: Supplementary file 1 [file DataSheet_1.pdf]

Lomerizine inhibits LPS-mediated neuroinflammation and tau hyperphosphorylation by modulating NLRP3 and DYRK1A/GSK3α/β

Jin-Hee Park[1,2,3]| Jeong-Woo Hwang[1,3]| Hyun-ju Lee[1,3]| Geum Mi Jang[2] | Yoo Joo Jeong[1,2] | Joonho Cho[2] | Jinsoo Seo[2,*] | Hyang-Sook Hoe[1,2,*]

[1]Department of Neural Development and Disease, Korea Brain Research Institute (KBRI), Daegu, Republic of Korea; [2]Department of Brain Sciences, Daegu Gyeongbuk Institute of Science & Technology, Daegu, Republic of Korea. [3]These authors contributed equally to this work.

*Corresponding author

Hyang-Sook Hoe, Ph.D.: Department of Neural Development and Disease, Korea Brain Research Institute (KBRI), 61 Cheomdan-ro, Dong-gu, Daegu, Korea, 41068; E-mail: sookhoe72@kbri.re.kr

Jinsoo Seo, Ph.D.: Department of Brain Sciences, Daegu Gyeongbuk Institute of Science & Technology, Daegu 42988; E-mail: jsseo@dgist.ac.kr

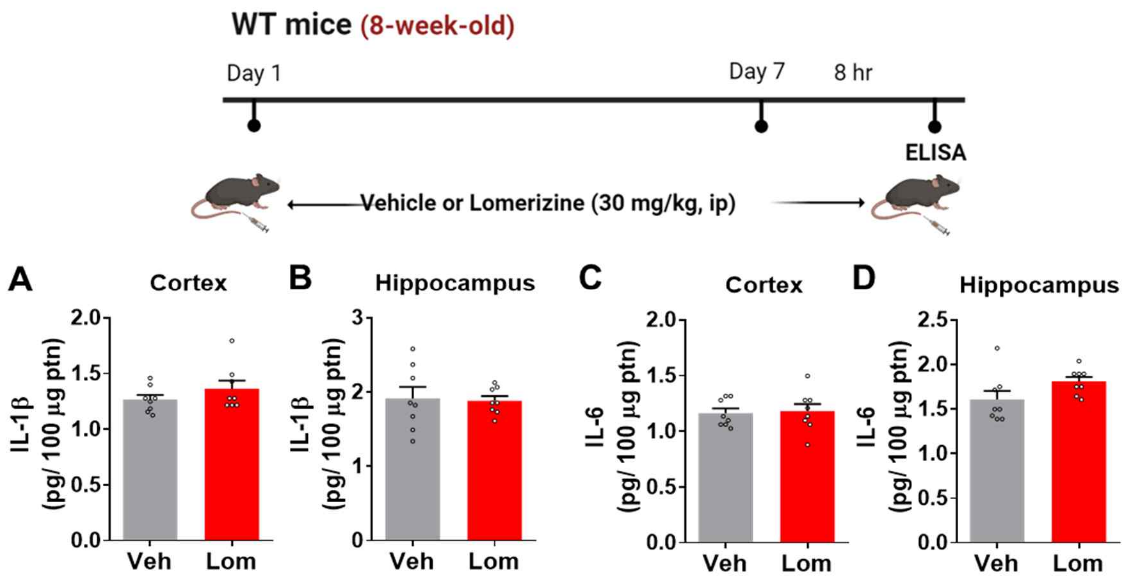

Supplementary Figure 1. Lomerizine alone does not alter proinflammatory cytokine levels in vivo. (A-D) Wild-type mice were injected with vehicle (2% DMSO) or lomerizine (30 mg/kg, i.p.) daily for 7 days, and the cortex and hippocampus were dissected. ELISA analysis of IL-1β and IL-6 levels in the cortex and hippocampus (n = 8/group).

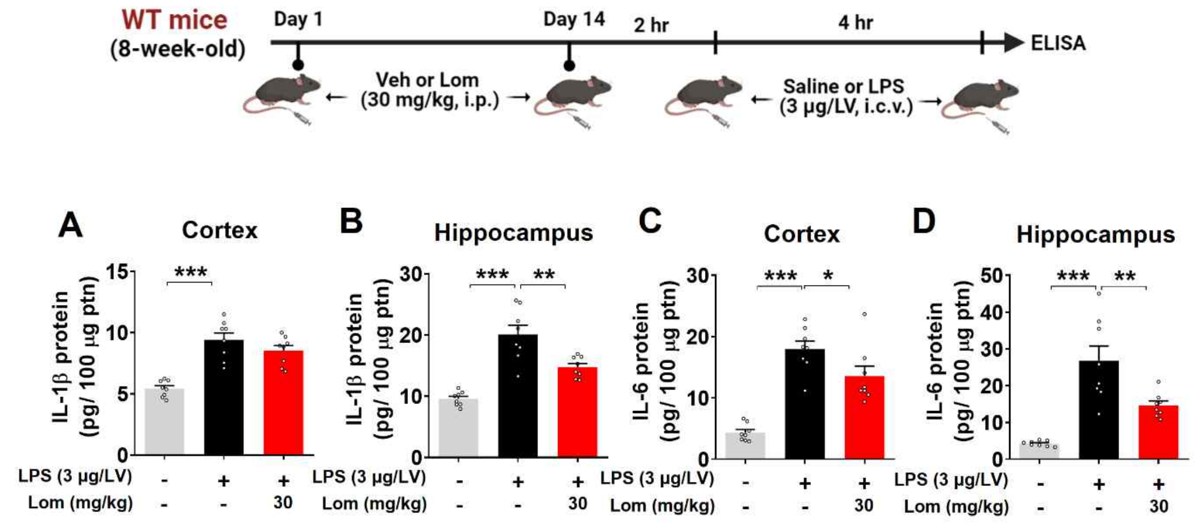

Supplementary Figure 2. Pretreatment with lomerizine reduces the induction of proinflammatory cytokine expression by i.c.v. injection of LPS in vivo. (A-D) Wild-type mice were injected with vehicle (2% DMSO) or lomerizine (30 mg/kg, i.p.) daily for 14 days. On day 14, LPS was injected i.c.v. 2 h after the last vehicle/lomerizine injection, respectively. Four hours after the LPS injection, the cortex and hippocampus were dissected. ELISA analysis of IL-1β and IL-6 levels in the cortex and hippocampus in wild-type mice treated for 14 days (n = 8/group). *$p < 0.05$, **$p < 0.01$, ***$p < 0.001$.

Supplementary table 1. Results of one-way ANOVA (Tukey's, Newman-Keuls and Sidak's tests) and the t-test to determine the statistical significance of the results of the in vitro and in vivo experiments.

**Figure 1B. MTT**						
Number of families	1					
Number of comparisons per family	45					
Alpha	0.05					
Tukey's multiple comparisons test	Mean Diff.	95.00% CI of diff.	Significant?	Summary	Adjusted P Value	
0.1 Veh vs. 0.1 mM	-2.974	-15.02 to 9.067	No	ns	0.9985	A-B
0.1 Veh vs. 1 Veh	-5.606e-006	-12.04 to 12.04	No	ns	>0.9999	A-C
0.1 Veh vs. 1 mM	-4.063	-16.1 to 7.979	No	ns	0.9847	A-D
0.1 Veh vs. 5 Veh	2.273e-007	-12.04 to 12.04	No	ns	>0.9999	A-E
0.1 Veh vs. 5 mM	-4.095	-16.14 to 7.947	No	ns	0.9839	A-F
0.1 Veh vs. 10 Veh	-8.106e-006	-12.04 to 12.04	No	ns	>0.9999	A-G
0.1 Veh vs. 10 mM	-14.04	-26.08 to -1.998	Yes	**	0.0097	A-H
0.1 Veh vs. 20 Veh	-8.182e-006	-12.3 to 12.3	No	ns	>0.9999	A-I
0.1 Veh vs. 20 mM	-1.638	-13.68 to 10.4	No	ns	>0.9999	A-J
0.1 mM vs. 1 Veh	2.974	-8.803 to 14.75	No	ns	0.9982	B-C
0.1 mM vs. 1 mM	-1.089	-12.87 to 10.69	No	ns	>0.9999	B-D
0.1 uM vs. 5 Veh	2.974	-8.803 to 14.75	No	ns	0.9982	B-E
0.1 mM vs. 5 mM	-1.12	-12.9 to 10.66	No	ns	>0.9999	B-F
0.1 mM vs. 10 Veh	2.974	-8.803 to 14.75	No	ns	0.9982	B-G
0.1 mM vs. 10 mM	-11.07	-22.84 to 0.7116	No	ns	0.0844	B-H
0.1 mM vs. 20 Veh	2.974	-9.067 to 15.02	No	ns	0.9985	B-I
0.1 mM vs. 20 mM	1.336	-10.44 to 13.11	No	ns	>0.9999	B-J
1 Veh vs. 1 mM	-4.063	-15.84 to 7.714	No	ns	0.9822	C-D
1 Veh vs. 5 Veh	5.833e-006	-11.78 to 11.78	No	ns	>0.9999	C-E
1 Veh vs. 5 mM	-4.095	-15.87 to 7.682	No	ns	0.9812	C-F
1 Veh vs. 10 Veh	-2.5e-006	-11.78 to 11.78	No	ns	>0.9999	C-G
1 Veh vs. 10 mM	-14.04	-25.82 to -2.263	Yes	**	0.0073	C-H
1 Veh vs. 20 Veh	-2.576e-006	-12.04 to 12.04	No	ns	>0.9999	C-I
1 Veh vs. 20 mM	-1.638	-13.42 to 10.14	No	ns	>0.9999	C-J
1 mM vs. 5 Veh	4.063	-7.714 to 15.84	No	ns	0.9822	D-E
1 mM vs. 5 mM	-0.0317	-11.81 to 11.75	No	ns	>0.9999	D-F
1 mM vs. 10 Veh	4.063	-7.714 to 15.84	No	ns	0.9822	D-G
1 mM vs. 10 mM	-9.977	-21.75 to 1.8	No	ns	0.1725	D-H
1 uM vs. 20 Veh	4.063	-7.979 to 16.1	No	ns	0.9847	D-I
1 mM vs. 20 mM	2.425	-9.352 to 14.2	No	ns	0.9996	D-J
5 Veh vs. 5 mM	-4.095	-15.87 to 7.682	No	ns	0.9812	E-F
5 Veh vs. 10 Veh	-8.333e-006	-11.78 to 11.78	No	ns	>0.9999	E-G
5 Veh vs. 10 mM	-14.04	-25.82 to -2.263	Yes	**	0.0073	E-H
5 Veh vs. 20 Veh	-8.409e-006	-12.04 to 12.04	No	ns	>0.9999	E-I
5 Veh vs. 20 mM	-1.638	-13.42 to 10.14	No	ns	>0.9999	E-J
5 mM vs. 10 Veh	4.095	-7.682 to 15.87	No	ns	0.9812	F-G
5 mM vs. 10 mM	-9.945	-21.72 to 1.832	No	ns	0.1758	F-H
5 mM vs. 20 Veh	4.095	-7.947 to 16.14	No	ns	0.9839	F-I
5 mM vs. 20 mM	2.457	-9.32 to 14.23	No	ns	0.9996	F-J
10 Veh vs. 10 uM	-14.04	-25.82 to -2.263	Yes	**	0.0073	G-H

10 Veh vs. 20 Veh	-7.576e-008	-12.04 to 12.04	No	ns	>0.9999	G-I
10 Veh vs. 20 mM	-1.638	-13.42 to 10.14	No	ns	>0.9999	G-J
10 mM vs. 20 Veh	14.04	1.998 to 26.08	Yes	**	0.0097	H-I
10 mM vs. 20 mM	12.4	0.6247 to 24.18	Yes	*	0.0305	H-J
20 Veh vs. 20 mM	-1.638	-13.68 to 10.4	No	ns	>0.9999	I-J

Figure 1C. COX-2

Number of families	1					
Number of comparisons per family	3					
Alpha	0.05					
Tukey's multiple comparisons test	Mean Diff.	95.00% CI of diff.	Significant?	Summary	Adjusted P Value	
Column A vs. Column B	-4.516	-7.126 to -1.905	Yes	***	0.0008	A-B
Column A vs. Column C	-2.462	-5.072 to 0.1486	No	ns	0.0668	A-C
Column B vs. Column C	2.054	-0.5565 to 4.665	No	ns	0.1411	B-C

Figure 1D. IL-1β

Number of families	1					
Number of comparisons per family	3					
Alpha	0.05					
Tukey's multiple comparisons test	Mean Diff.	95.00% CI of diff.	Significant?	Summary	Adjusted P Value	
Column A vs. Column B	-18.01	-26.04 to -9.979	Yes	****	<0.0001	A-B
Column A vs. Column C	-2.392	-10.42 to 5.638	No	ns	0.7365	A-C
Column B vs. Column C	15.62	7.587 to 23.65	Yes	***	0.0002	B-C

Figure 1E. Pro-IL-1β

Number of families	1					
Number of comparisons per family	3					
Alpha	0.05					
Tukey's multiple comparisons test	Mean Diff.	95.00% CI of diff.	Significant?	Summary	Adjusted P Value	
Column A vs. Column B	-14.44	-17.34 to -11.55	Yes	****	<0.0001	A-B
Column A vs. Column C	-2.553	-5.450 to 0.3443	No	ns	0.0907	A-C
Column B vs. Column C	11.89	8.992 to 14.79	Yes	****	<0.0001	B-C

Figure 1F. IL-6

Number of families	1					
Number of comparisons per family	3					
Alpha	0.05					
Tukey's multiple comparisons test	Mean Diff.	95.00% CI of diff.	Significant?	Summary	Adjusted P Value	
Column A vs. Column B	-7.026	-9.814 to -4.239	Yes	****	<0.0001	A-B
Column A vs. Column C	-3.704	-6.492 to -0.9168	Yes	**	0.0082	A-C
Column B vs. Column C	3.322	0.5341 to 6.110	Yes	*	0.0178	B-C

Figure 1G. TNF-α

Number of families	1					
Number of comparisons per family	3					
Alpha	0.05					
Tukey's multiple comparisons test	Mean Diff.	95.00% CI of diff.	Significant?	Summary	Adjusted P Value	
Column A vs. Column B	-15.97	-25.36 to -6.573	Yes	***	0.0009	A-B
Column A vs. Column C	-8.334	-17.73 to 1.058	No	ns	0.0880	A-C
Column B vs. Column C	7.631	-1.761 to 17.02	No	ns	0.1255	B-C

Figure 1H. CDK6

Number of families	1					
Number of comparisons per family	3					
Alpha	0.05					
Tukey's multiple comparisons test	Mean Diff.	95.00% CI of diff.	Significant?	Summary	Adjusted P Value	
Column A vs. Column B	-0.6777	-1.313 to -0.04232	Yes	*	0.0352	A-B
Column A vs. Column C	-0.5401	-1.175 to 0.09531	No	ns	0.1053	A-C
Column B vs. Column C	0.1376	-0.4977 to 0.7730	No	ns	0.8496	B-C

Figure 1I. NLRP3

Number of families	1
Number of comparisons per family	3

Alpha	0.05					
Tukey's multiple comparisons test	Mean Diff.	95.00% CI of diff.	Significant?	Summary	Adjusted P Value	
Column A vs. Column B	-2.540	-3.267 to -1.814	Yes	****	<0.0001	A-B
Column A vs. Column C	-1.493	-2.219 to -0.7661	Yes	***	0.0001	A-C
Column B vs. Column C	1.048	0.3211 to 1.774	Yes	**	0.0042	B-C
**Figure 1J. SOD2**						
Number of families	1					
Number of comparisons per family	3					
Alpha	0.05					
Tukey's multiple comparisons test	Mean Diff.	95.00% CI of diff.	Significant?	Summary	Adjusted P Value	
Column A vs. Column B	-0.8716	-1.378 to -0.3651	Yes	***	0.0008	A-B
Column A vs. Column C	-0.4224	-0.9289 to 0.08401	No	ns	0.1135	A-C
Column B vs. Column C	0.4491	-0.05731 to 0.9556	No	ns	0.0883	B-C
**Figure 2B. Iba-1 fluorescence intensity - Cortex**						
Number of families	1					
Number of comparisons per family	6					
Alpha	0.05					
Tukey's multiple comparisons test	Mean Diff.	95.00% CI of diff.	Significant?	Summary	Adjusted P Value	
Column A vs. Column B	-145.3	-199.6 to -90.98	Yes	****	<0.0001	A-B
Column A vs. Column C	-114.4	-168.7 to -60.04	Yes	****	<0.0001	A-C
Column A vs. Column D	-39.68	-94 to 14.64	No	ns	0.2287	A-D
Column B vs. Column C	30.94	-21.93 to 83.81	No	ns	0.4202	B-C
Column B vs. Column D	105.6	52.76 to 158.5	Yes	****	<0.0001	B-D
Column C vs. Column D	74.68	21.81 to 127.6	Yes	**	0.0022	C-D
**Figure 2C. Iba-1 fluorescence intensity - CA1**						
Number of families	1					
Number of comparisons per family	6					
Alpha	0.05					
Tukey's multiple comparisons test	Mean Diff.	95.00% CI of diff.	Significant?	Summary	Adjusted P Value	
Column A vs. Column B	-47.41	-63.38 to -31.43	Yes	****	<0.0001	A-B
Column A vs. Column C	-32.49	-48.46 to -16.51	Yes	****	<0.0001	A-C
Column A vs. Column D	-18.17	-34.15 to -2.196	Yes	*	0.0194	A-D
Column B vs. Column C	14.92	-0.8489 to 30.69	No	ns	0.0703	B-C
Column B vs. Column D	29.23	13.46 to 45.00	Yes	****	<0.0001	B-D
Column C vs. Column D	14.31	-1.456 to 30.08	No	ns	0.0888	C-D
**Figure 2D. Iba-1 fluorescence intensity - DG**						
Number of families	1					
Number of comparisons per family	6					
Alpha	0.05					
Tukey's multiple comparisons test	Mean Diff.	95.00% CI of diff.	Significant?	Summary	Adjusted P Value	
Column A vs. Column B	-55.61	-74.40 to -36.83	Yes	****	<0.0001	A-B
Column A vs. Column C	-39.57	-58.35 to -20.78	Yes	****	<0.0001	A-C
Column A vs. Column D	-26.67	-45.46 to -7.883	Yes	**	0.0021	A-D
Column B vs. Column C	16.05	-2.498 to 34.59	No	ns	0.1135	B-C
Column B vs. Column D	28.94	10.40 to 47.49	Yes	***	0.0006	B-D
Column C vs. Column D	12.89	-5.652 to 31.44	No	ns	0.2690	C-D
**Figure 2E. Iba-1 positive area - Cortex**						
Number of families	1					
Number of comparisons per family	6					
Alpha	0.05					
Tukey's multiple comparisons test	Mean Diff.	95.00% CI of diff.	Significant?	Summary	Adjusted P Value	
Column A vs. Column B	-1.584	-2.003 to -1.165	Yes	****	<0.0001	A-B
Column A vs. Column C	-1.422	-1.841 to -1.003	Yes	****	<0.0001	A-C
Column A vs. Column D	-0.5118	-0.9311 to -0.09248	Yes	*	0.0104	A-D
Column B vs. Column C	0.1621	-0.246 to 0.5702	No	ns	0.7243	B-C
Column B vs. Column D	1.072	0.6643 to 1.48	Yes	****	<0.0001	B-D
Column C vs. Column D	0.9103	0.5022 to 1.318	Yes	****	<0.0001	C-D

Figure 2F. Iba-1 positive area - CA1							
Number of families	1						
Number of comparisons per family	6						
Alpha	0.05						
Tukey's multiple comparisons test	Mean Diff.	95.00% CI of diff.	Significant?	Summary	Adjusted P Value		
Column A vs. Column B	-3.941	-5.2 to -2.682	Yes	****	<0.0001	A-B	
Column A vs. Column C	-2.951	-4.21 to -1.692	Yes	****	<0.0001	A-C	
Column A vs. Column D	-1.431	-2.69 to -0.1717	Yes	*	0.0195	A-D	
Column B vs. Column C	0.9908	-0.252 to 2.234	No	ns	0.1642	B-C	
Column B vs. Column D	2.511	1.268 to 3.753	Yes	****	<0.0001	B-D	
Column C vs. Column D	1.52	0.2771 to 2.763	Yes	*	0.0102	C-D	
**Figure 2G. Iba-1 positive area - DG**							
Number of families	1						
Number of comparisons per family	6						
Alpha	0.05						
Tukey's multiple comparisons test	Mean Diff.	95.00% CI of diff.	Significant?	Summary	Adjusted P Value		
Column A vs. Column B	-4.259	-5.478 to -3.04	Yes	****	<0.0001	A-B	
Column A vs. Column C	-3.205	-4.424 to -1.986	Yes	****	<0.0001	A-C	
Column A vs. Column D	-1.778	-2.997 to -0.5585	Yes	**	0.0015	A-D	
Column B vs. Column C	1.054	-0.1498 to 2.257	No	ns	0.1071	B-C	
Column B vs. Column D	2.481	1.278 to 3.685	Yes	****	<0.0001	B-D	
Column C vs. Column D	1.428	0.2242 to 2.631	Yes	*	0.0135	C-D	
**Figure 2H. Iba-1 positive cells/mm^2 - Cortex**							
Number of families	1						
Number of comparisons per family	6						
Alpha	0.05						
Tukey's multiple comparisons test	Mean Diff.	95.00% CI of diff.	Significant?	Summary	Adjusted P Value		
Column A vs. Column B	-261.1	-344.4 to -177.8	Yes	****	<0.0001	A-B	
Column A vs. Column C	-245.9	-329.2 to -162.6	Yes	****	<0.0001	A-C	
Column A vs. Column D	-97.08	-180.4 to -13.77	Yes	*	0.0158	A-D	
Column B vs. Column C	15.19	-65.91 to 96.28	No	ns	0.9606	B-C	
Column B vs. Column D	164	82.93 to 245.1	Yes	****	<0.0001	B-D	
Column C vs. Column D	148.8	67.74 to 229.9	Yes	****	<0.0001	C-D	
**Figure 2I. Iba-1 positive cells/mm^2 - CA1**							
Number of families	1						
Number of comparisons per family	6						
Alpha	0.05						
Tukey's multiple comparisons test	Mean Diff.	95.00% CI of diff.	Significant?	Summary	Adjusted P Value		
Column F vs. Column G	-521.5	-737.2 to -305.8	Yes	****	<0.0001	A-B	
Column F vs. Column H	-482	-697.7 to -266.3	Yes	****	<0.0001	A-C	
Column F vs. Column I	-255.2	-470.9 to -39.47	Yes	*	0.0139	A-D	
Column G vs. Column H	39.51	-173.4 to 252.5	No	ns	0.9617	B-C	
Column G vs. Column I	266.3	53.37 to 479.3	Yes	**	0.0082	B-D	
Column H vs. Column I	226.8	13.86 to 439.8	Yes	*	0.0324	C-D	
**Figure 2J. Iba-1 positive cells/mm^2 - DG**							
Number of families	1						
Number of comparisons per family	6						
Alpha	0.05						
Tukey's multiple comparisons test	Mean Diff.	95.00% CI of diff.	Significant?	Summary	Adjusted P Value		
Column A vs. Column B	-456.5	-624.8 to -288.2	Yes	****	<0.0001	A-B	
Column A vs. Column C	-437.2	-605.5 to -268.9	Yes	****	<0.0001	A-C	
Column A vs. Column D	-339	-507.3 to -170.7	Yes	****	<0.0001	A-D	
Column B vs. Column C	19.28	-146.9 to 185.4	No	ns	0.9901	B-C	
Column B vs. Column D	117.5	-48.62 to 283.7	No	ns	0.2547	B-D	
Column C vs. Column D	98.23	-67.9 to 264.4	No	ns	0.4112	C-D	
**Figure 3B. GFAP fluorescence intensity - Cortex**							
Number of families	1						

Number of comparisons per family	6					
Alpha	0.05					
Tukey's multiple comparisons test	Mean Diff.	95.00% CI of diff.	Significant?	Summary	Adjusted P Value	
Column A vs. Column B	-1794	-2368 to -1220	Yes	****	<0.0001	A-B
Column A vs. Column C	-1244	-1818 to -669.7	Yes	****	<0.0001	A-C
Column A vs. Column D	-553.3	-1127 to 20.64	No	ns	0.0629	A-D
Column B vs. Column C	550.5	0.3282 to 1101	Yes	*	0.0498	B-C
Column B vs. Column D	1241	690.7 to 1791	Yes	****	<0.0001	B-D
Column C vs. Column D	690.3	140.2 to 1241	Yes	**	0.0080	C-D
**Figure 3C. GFAP fluorescence intensity - CA1**						
Number of families	1					
Number of comparisons per family	6					
Alpha	0.05					
Tukey's multiple comparisons test	Mean Diff.	95.00% CI of diff.	Significant?	Summary	Adjusted P Value	
Column A vs. Column B	-309	-427.8 to -190.1	Yes	****	<0.0001	A-B
Column A vs. Column C	-178.9	-297.8 to -60.01	Yes	***	0.0010	A-C
Column A vs. Column D	-53.36	-172.2 to 65.52	No	ns	0.6415	A-D
Column B vs. Column C	130.1	14.35 to 245.8	Yes	*	0.0214	B-C
Column B vs. Column D	255.6	139.9 to 371.3	Yes	****	<0.0001	B-D
Column C vs. Column D	125.5	9.822 to 241.2	Yes	*	0.0282	C-D
**Figure 3D. GFAP fluorescence intensity - DG**						
Number of families	1					
Number of comparisons per family	6					
Alpha	0.05					
Tukey's multiple comparisons test	Mean Diff.	95.00% CI of diff.	Significant?	Summary	Adjusted P Value	
Column A vs. Column B	-285.1	-397.2 to -173	Yes	****	<0.0001	A-B
Column A vs. Column C	-190.1	-302.3 to -78.04	Yes	***	0.0002	A-C
Column A vs. Column D	-81.83	-193.9 to 30.28	No	ns	0.2292	A-D
Column B vs. Column C	94.95	-14.16 to 204.1	No	ns	0.1103	B-C
Column B vs. Column D	203.3	94.16 to 312.4	Yes	****	<0.0001	B-D
Column C vs. Column D	108.3	-0.7942 to 217.4	No	ns	0.0524	C-D
**Figure 3E. GFAP positive area- Cortex**						
Number of families	1					
Number of comparisons per family	6					
Alpha	0.05					
Tukey's multiple comparisons test	Mean Diff.	95.00% CI of diff.	Significant?	Summary	Adjusted P Value	
Column A vs. Column B	-0.3224	-0.5334 to -0.1113	Yes	***	0.0008	A-B
Column A vs. Column C	-0.2219	-0.4305 to -0.01339	Yes	*	0.0326	A-C
Column A vs. Column D	-0.188	-0.3965 to 0.02057	No	ns	0.0920	A-D
Column B vs. Column C	0.1004	-0.1021 to 0.303	No	ns	0.5634	B-C
Column B vs. Column D	0.1344	-0.06816 to 0.3369	No	ns	0.3084	B-D
Column C vs. Column D	0.03396	-0.166 to 0.2339	No	ns	0.9701	C-D
**Figure 3F. GFAP positive area - CA1**						
Number of families	1					
Number of comparisons per family	6					
Alpha	0.05					
Tukey's multiple comparisons test	Mean Diff.	95.00% CI of diff.	Significant?	Summary	Adjusted P Value	
Column A vs. Column B	-5.612	-8.387 to -2.837	Yes	****	<0.0001	A-B
Column A vs. Column C	-1.907	-4.717 to 0.903	No	ns	0.2893	A-C
Column A vs. Column D	-0.3343	-3.109 to 2.44	No	ns	0.9889	A-D
Column B vs. Column C	3.705	0.9302 to 6.48	Yes	**	0.0042	B-C
Column B vs. Column D	5.278	2.539 to 8.016	Yes	****	<0.0001	B-D
Column C vs. Column D	1.573	-1.202 to 4.347	No	ns	0.4488	C-D
**Figure 3G. GFAP positive area - DG**						
Number of families	1					
Number of comparisons per family	6					
Alpha	0.05					

Tukey's multiple comparisons test	Mean Diff.	95.00% CI of diff.	Significant?	Summary	Adjusted P Value	
Column A vs. Column B	-4.426	-6.754 to -2.098	Yes	****	<0.0001	A-B
Column A vs. Column C	-1.877	-4.233 to 0.4802	No	ns	0.1649	A-C
Column A vs. Column D	-0.7318	-3.06 to 1.596	No	ns	0.8417	A-D
Column B vs. Column C	2.549	0.2537 to 4.845	Yes	*	0.0236	B-C
Column B vs. Column D	3.694	1.428 to 5.96	Yes	***	0.0003	B-D
Column C vs. Column D	1.145	-1.151 to 3.44	No	ns	0.5588	C-D

Figure 3H. GFAP positive cells/mm^2 - Cortex

Number of families	1					
Number of comparisons per family	6					
Alpha	0.05					
Tukey's multiple comparisons test	Mean Diff.	95.00% CI of diff.	Significant?	Summary	Adjusted P Value	
Column A vs. Column B	-69.03	-104.6 to -33.44	Yes	****	<0.0001	A-B
Column A vs. Column C	-45.76	-80.91 to -10.60	Yes	**	0.0055	A-C
Column A vs. Column D	-28.03	-63.19 to 7.120	No	ns	0.1639	A-D
Column B vs. Column C	23.27	-11.39 to 57.93	No	ns	0.2983	B-C
Column B vs. Column D	40.99	6.332 to 75.66	Yes	*	0.0139	B-D
Column C vs. Column D	17.72	-16.49 to 51.94	No	ns	0.5271	C-D

Figure 3I. GFAP positive cells/mm^2 - CA1

Number of families	1					
Number of comparisons per family	6					
Alpha	0.05					
Tukey's multiple comparisons test	Mean Diff.	95.00% CI of diff.	Significant?	Summary	Adjusted P Value	
Column A vs. Column B	-550.4	-769.8 to -331	Yes	****	<0.0001	A-B
Column A vs. Column C	-341.4	-560.8 to -121.9	Yes	***	0.0006	A-C
Column A vs. Column D	-101.2	-320.6 to 118.3	No	ns	0.6217	A-D
Column B vs. Column C	209	-7.559 to 425.6	No	ns	0.0625	B-C
Column B vs. Column D	449.2	232.7 to 665.8	Yes	****	<0.0001	B-D
Column C vs. Column D	240.2	23.62 to 456.8	Yes	*	0.0238	C-D

Figure 3J. GFAP positive cells/mm^2- DG

Number of families	1					
Number of comparisons per family	6					
Alpha	0.05					
Tukey's multiple comparisons test	Mean Diff.	95.00% CI of diff.	Significant?	Summary	Adjusted P Value	
Column A vs. Column B	-580	-821.3 to -338.6	Yes	****	<0.0001	A-B
Column A vs. Column C	-397.3	-638.7 to -156	Yes	***	0.0003	A-C
Column A vs. Column D	-166.2	-407.6 to 75.16	No	ns	0.2769	A-D
Column B vs. Column C	182.6	-52.3 to 417.6	No	ns	0.1817	B-C
Column B vs. Column D	413.8	178.8 to 648.7	Yes	****	<0.0001	B-D
Column C vs. Column D	231.1	-3.814 to 466.1	No	ns	0.0555	C-D

Figure 4B. IL-6 fluorescence intensity - Cortex

Number of families	1					
Number of comparisons per family	6					
Alpha	0.05					
Tukey's multiple comparisons test	Mean Diff.	95.00% CI of diff.	Significant?	Summary	Adjusted P Value	
Column A vs. Column B	-46.38	-73.36 to -19.39	Yes	***	0.0001	A-B
Column A vs. Column C	-50.55	-77.85 to -23.25	Yes	****	<0.0001	A-C
Column A vs. Column D	0.8483	-26.45 to 28.15	No	ns	0.9998	A-D
Column B vs. Column C	-4.177	-30.8 to 22.45	No	ns	0.9762	B-C
Column B vs. Column D	47.22	20.6 to 73.85	Yes	****	<0.0001	B-D
Column C vs. Column D	51.4	24.45 to 78.35	Yes	****	<0.0001	C-D

Figure 4C. IL-6 fluorescence intensity - CA1

Number of families	1					
Number of comparisons per family	6					
Alpha	0.05					
Tukey's multiple comparisons test	Mean Diff.	95.00% CI of diff.	Significant?	Summary	Adjusted P Value	
Column A vs. Column B	-49.47	-71.98 to -26.96	Yes	****	<0.0001	A-B

Column A vs. Column C	-50.14	-72.9 to -27.37	Yes	****	<0.0001	A-C
Column A vs. Column D	-4.952	-27.72 to 17.81	No	ns	0.9408	A-D
Column B vs. Column C	-0.6705	-23.43 to 22.09	No	ns	0.9998	B-C
Column B vs. Column D	44.52	21.75 to 67.28	Yes	****	<0.0001	B-D
Column C vs. Column D	45.19	22.17 to 68.2	Yes	****	<0.0001	C-D
**Figure 4D. IL-6 fluorescence intensity - DG**						
Number of families	1					
Number of comparisons per family	6					
Alpha	0.05					
Tukey's multiple comparisons test	Mean Diff.	95.00% CI of diff.	Significant?	Summary	Adjusted P Value	
Column A vs. Column B	-49.03	-72.34 to -25.73	Yes	****	<0.0001	A-B
Column A vs. Column C	-49.67	-73.23 to -26.1	Yes	****	<0.0001	A-C
Column A vs. Column D	-8.121	-31.69 to 15.44	No	ns	0.8033	A-D
Column B vs. Column C	-0.6325	-24.2 to 22.93	No	ns	0.9999	B-C
Column B vs. Column D	40.91	17.35 to 64.48	Yes	***	0.0001	B-D
Column C vs. Column D	41.54	17.72 to 65.37	Yes	****	<0.0001	C-D
**Figure 4E. IL-6 ELISA – Cortex**						
Number of families	1					
Number of comparisons per family	3					
Alpha	0.05					
Tukey's multiple comparisons test	Mean Diff.	95.00% CI of diff.	Significant?	Summary	Adjusted P Value	
Column A vs. Column B	-39.22	-60.10 to -18.34	Yes	***	0.0003	A-B
Column A vs. Column C	-27.97	-48.85 to -7.095	Yes	**	0.0077	A-C
Column B vs. Column C	11.25	-9.630 to 32.13	No	ns	0.3803	B-C
**Figure 4F. IL-6 ELISA - Hippocampus**						
Number of families	1					
Number of comparisons per family	3					
Alpha	0.05					
Holm-Sidak's multiple comparisons test	Mean Diff.		Significant?	Summary	Adjusted P Value	
Column A vs. Column B	-31.44		Yes	***	0.0002	A-B
Column A vs. Column C	-17.10		Yes	*	0.0249	A-C
Column B vs. Column C	14.34		Yes	*	0.0326	B-C
**Figure 5B. NLRP3 fluorescence intensity - Cortex**						
Number of families	1					
Number of comparisons per family	6					
Alpha	0.05					
Tukey's multiple comparisons test	Mean Diff.	95.00% CI of diff.	Significant?	Summary	Adjusted P Value	
Column A vs. Column B	-20.98	-37.65 to -4.302	Yes	**	0.0078	A-B
Column A vs. Column C	-24.31	-40.99 to -7.638	Yes	**	0.0015	A-C
Column A vs. Column D	8.596	-7.868 to 25.06	No	ns	0.5203	A-D
Column B vs. Column C	-3.336	-20.01 to 13.34	No	ns	0.9525	B-C
Column B vs. Column D	29.57	13.11 to 46.04	Yes	****	<0.0001	B-D
Column C vs. Column D	32.91	16.44 to 49.37	Yes	****	<0.0001	C-D
**Figure 5C. NLRP3 fluorescence intensity - CA1**						
Number of families	1					
Number of comparisons per family	6					
Alpha	0.05					
Tukey's multiple comparisons test	Mean Diff.	95.00% CI of diff.	Significant?	Summary	Adjusted P Value	
Column A vs. Column B	-19.08	-33.03 to -5.123	Yes	**	0.0032	A-B
Column A vs. Column C	-11.06	-25.2 to 3.07	No	ns	0.1771	A-C
Column A vs. Column D	0.7682	-12.86 to 14.4	No	ns	0.9988	A-D
Column B vs. Column C	8.011	-6.123 to 22.15	No	ns	0.4494	B-C
Column B vs. Column D	19.84	6.212 to 33.47	Yes	**	0.0015	B-D
Column C vs. Column D	11.83	-1.986 to 25.65	No	ns	0.1195	C-D
**Figure 5D. NLRP3 fluorescence intensity - DG**						
Number of families	1					
Number of comparisons per family	6					

Alpha	0.05					
Tukey's multiple comparisons test	Mean Diff.	95.00% CI of diff.	Significant?	Summary	Adjusted P Value	
Column A vs. Column B	-25.23	-39.55 to -10.91	Yes	****	<0.0001	A-B
Column A vs. Column C	-12.62	-27.12 to 1.89	No	ns	0.1107	A-C
Column A vs. Column D	-1.989	-15.98 to 12	No	ns	0.9821	A-D
Column B vs. Column C	12.61	-1.896 to 27.12	No	ns	0.1110	B-C
Column B vs. Column D	23.24	9.249 to 37.23	Yes	***	0.0002	B-D
Column C vs. Column D	10.63	-3.554 to 24.81	No	ns	0.2091	C-D
**Figure 5F. IL-1β fluorescence intensity - Cortex**						
Number of families	1					
Number of comparisons per family	6					
Alpha	0.05					
Tukey's multiple comparisons test	Mean Diff.	95.00% CI of diff.	Significant?	Summary	Adjusted P Value	
Column A vs. Column B	-54.96	-74.81 to -35.1	Yes	****	<0.0001	A-B
Column A vs. Column C	-56.87	-76.73 to -37.01	Yes	****	<0.0001	A-C
Column A vs. Column D	-21.04	-40.9 to -1.182	Yes	*	0.0336	A-D
Column B vs. Column C	-1.912	-21.77 to 17.95	No	ns	0.9943	B-C
Column B vs. Column D	33.92	14.06 to 53.77	Yes	***	0.0001	B-D
Column C vs. Column D	35.83	15.97 to 55.69	Yes	****	<0.0001	C-D
**Figure 5G. IL-1β fluorescence intensity - CA1**						
Number of families	1					
Number of comparisons per family	6					
Alpha	0.05					
Tukey's multiple comparisons test	Mean Diff.	95.00% CI of diff.	Significant?	Summary	Adjusted P Value	
Column A vs. Column B	-37.83	-52.7 to -22.97	Yes	****	<0.0001	A-B
Column A vs. Column C	-31.25	-46.11 to -16.39	Yes	****	<0.0001	A-C
Column A vs. Column D	-14.13	-28.99 to 0.7332	No	ns	0.0684	A-D
Column B vs. Column C	6.584	-8.278 to 21.45	No	ns	0.6514	B-C
Column B vs. Column D	23.71	8.845 to 38.57	Yes	***	0.0004	B-D
Column C vs. Column D	17.12	2.261 to 31.98	Yes	*	0.0174	C-D
**Figure 5H. IL-1β fluorescence intensity - DG**						
Number of families	1					
Number of comparisons per family	6					
Alpha	0.05					
Tukey's multiple comparisons test	Mean Diff.	95.00% CI of diff.	Significant?	Summary	Adjusted P Value	
Column A vs. Column B	-45.49	-61.73 to -29.26	Yes	****	<0.0001	A-B
Column A vs. Column C	-32.09	-48.32 to -15.86	Yes	****	<0.0001	A-C
Column A vs. Column D	-18.98	-35.21 to -2.747	Yes	*	0.0153	A-D
Column B vs. Column C	13.4	-2.829 to 29.63	No	ns	0.1413	B-C
Column B vs. Column D	26.51	10.28 to 42.75	Yes	***	0.0003	B-D
Column C vs. Column D	13.11	-3.12 to 29.34	No	ns	0.1555	C-D
**Figure 5I. IL-1β ELISA - Cortex**						
Number of families	1					
Number of comparisons per family	3					
Alpha	0.05					
Newman-Keuls multiple comparisons test	Mean Diff.		Significant?	Summary		
Column A vs. Column B	-23.20		Yes	***		A-B
Column A vs. Column C	-11.37		Yes	*		A-C
Column B vs. Column C	11.83		Yes	*		B-C
**Figure 5J. IL-1β ELISA - Hippocampus**						
Number of families	1					
Number of comparisons per family	3					
Alpha	0.05					
Tukey's multiple comparisons test	Mean Diff.	95.00% CI of diff.	Significant?	Summary	Adjusted P Value	
Column A vs. Column B	-22.05	-36.08 to -8.011	Yes	**	0.0020	A-B
Column A vs. Column C	-6.321	-20.36 to 7.714	No	ns	0.5038	A-C
Column B vs. Column C	15.72	1.690 to 29.76	Yes	*	0.0264	B-C

**Figure 5K-L. NLRP3 Western blot BV2 cell Vehicle vs LPS**	
Table Analyzed	Data 1
Column A	Data Set-A
vs.	vs.
Column B	Data Set-B
Ratio paired t test	
P value	0.0454
P value summary	*
Significantly different (P < 0.05)?	Yes
One- or two-tailed P value?	Two-tailed
t, df	t=2.368, df=8
Number of pairs	9

**Figure 5K-L. NLRP3 Western blot BV2 cell LPS vs lomerizine**	
Table Analyzed	Data 1
Column C	Data Set-C
vs.	vs.
Column B	Data Set-B
Paired t test	
P value	0.0237
P value summary	*
Significantly different (P < 0.05)?	Yes
One- or two-tailed P value?	Two-tailed
t, df	t=2.786, df=8
Number of pairs	9

Figure 5M. IL-1β ELISA BV2 cell

Number of families	1					
Number of comparisons per family	3					
Alpha	0.05					
Tukey's multiple comparisons test	Mean Diff.	95.00% CI of diff.	Significant?	Summary	Adjusted P Value	
Column A vs. Column B	-14.05	-18.53 to -9.574	Yes	****	<0.0001	A-B
Column A vs. Column C	-6.496	-10.97 to -2.019	Yes	**	0.0037	A-C
Column B vs. Column C	7.555	3.078 to 12.03	Yes	***	0.0009	B-C

Figure 6A. COX-2

Number of families	1					
Number of comparisons per family	3					
Alpha	0.05					
Tukey's multiple comparisons test	Mean Diff.	95.00% CI of diff.	Significant?	Summary	Adjusted P Value	
Column A vs. Column B	-69.78	-91.43 to -48.13	Yes	****	<0.0001	A-B
Column A vs. Column C	-51.78	-73.43 to -30.12	Yes	****	<0.0001	A-C
Column B vs. Column C	18.00	-2.801 to 38.81	No	ns	0.0963	B-C

Figure 6B. IL-1β

Number of families	1					
Number of comparisons per family	3					
Alpha	0.05					
Tukey's multiple comparisons test	Mean Diff.	95.00% CI of diff.	Significant?	Summary	Adjusted P Value	
Column A vs. Column B	-1412	-1988 to -835.8	Yes	****	<0.0001	A-B
Column A vs. Column C	-711.1	-1270 to -151.6	Yes	*	0.0119	A-C
Column B vs. Column C	701.1	165.0 to 1237	Yes	**	0.0098	B-C

Figure 6C. IL-6

Number of families	1					
Number of comparisons per family	3					
Alpha	0.05					
Tukey's multiple comparisons test	Mean Diff.	95.00% CI of diff.	Significant?	Summary	Adjusted P Value	
Column A vs. Column B	-44.07	-64.57 to -23.57	Yes	****	<0.0001	A-B
Column A vs. Column C	-36.12	-56.02 to -16.23	Yes	***	0.0006	A-C
Column B vs. Column C	7.946	-11.12 to 27.01	No	ns	0.5480	B-C

Figure 6D. TNF-α

Number of families	1					
Number of comparisons per family	3					
Alpha	0.05					
Tukey's multiple comparisons test	Mean Diff.	95.00% CI of diff.	Significant?	Summary	Adjusted P Value	
Column A vs. Column B	-39.42	-46.14 to -32.69	Yes	****	<0.0001	A-B
Column A vs. Column C	-30.01	-36.74 to -23.28	Yes	****	<0.0001	A-C

Column B vs. Column C	9.408	3.179 to 15.64	Yes	**	0.0030	B-C
**Figure 6E. CDK6**						
Number of families	1					
Number of comparisons per family	3					
Alpha	0.05					
Tukey's multiple comparisons test	Mean Diff.	95.00% CI of diff.	Significant?	Summary	Adjusted P Value	
Column A vs. Column B	-2.664	-3.131 to -2.198	Yes	****	<0.0001	A-B
Column A vs. Column C	-2.853	-3.320 to -2.387	Yes	****	<0.0001	A-C
Column B vs. Column C	-0.1890	-0.6211 to 0.2431	No	ns	0.5189	B-C
**Figure 6F. NLRP3**						
Number of families	1					
Number of comparisons per family	3					
Alpha	0.05					
Tukey's multiple comparisons test	Mean Diff.	95.00% CI of diff.	Significant?	Summary	Adjusted P Value	
Column A vs. Column B	-5.236	-6.688 to -3.784	Yes	****	<0.0001	A-B
Column A vs. Column C	-3.877	-5.329 to -2.425	Yes	****	<0.0001	A-C
Column B vs. Column C	1.359	-0.04363 to 2.762	No	ns	0.0586	B-C
**Figure 6G. SOD2**						
Number of families	1					
Number of comparisons per family	3					
Alpha	0.05					
Tukey's multiple comparisons test	Mean Diff.	95.00% CI of diff.	Significant?	Summary	Adjusted P Value	
Column A vs. Column B	-2.071	-3.313 to -0.8289	Yes	**	0.0012	A-B
Column A vs. Column C	-0.6193	-1.862 to 0.6230	No	ns	0.4326	A-C
Column B vs. Column C	1.452	0.2517 to 2.652	Yes	*	0.0163	B-C
**Figure 6H. IL-1β – Cortex**						
Number of families	1					
Number of comparisons per family	3					
Alpha	0.05					
Tukey's multiple comparisons test	Mean Diff.	95.00% CI of diff.	Significant?	Summary	Adjusted P Value	
Column A vs. Column B	-36.75	-45.81 to -27.70	Yes	****	<0.0001	A-B
Column A vs. Column C	-15.37	-24.75 to -5.999	Yes	**	0.0014	A-C
Column B vs. Column C	21.38	12.01 to 30.76	Yes	****	<0.0001	B-C
**Figure 6I. IL-1β – Hippocampus**						
Number of families	1					
Number of comparisons per family	3					
Alpha	0.05					
Tukey's multiple comparisons test	Mean Diff.	95.00% CI of diff.	Significant?	Summary	Adjusted P Value	
Column A vs. Column B	-32.39	-48.55 to -16.24	Yes	***	0.0002	A-B
Column A vs. Column C	-21.76	-36.71 to -6.802	Yes	**	0.0042	A-C
Column B vs. Column C	10.63	-5.519 to 26.79	No	ns	0.2413	B-C
**Figure 6J. TNF-α – Cortex**						
Number of families	1					
Number of comparisons per family	3					
Alpha	0.05					
Tukey's multiple comparisons test	Mean Diff.	95.00% CI of diff.	Significant?	Summary	Adjusted P Value	
Vehicle vs. LPS	-159.1	-211.0 to -107.2	Yes	****	<0.0001	A-B
Vehicle vs. Column C	-69.84	-121.7 to -17.94	Yes	**	0.0076	A-C
LPS vs. Column C	89.29	39.16 to 139.4	Yes	***	0.0006	B-C
**Figure 6K. TNF-α – Hippocampus**						
Number of families	1					
Number of comparisons per family	3					
Alpha	0.05					
Tukey's multiple comparisons test	Mean Diff.	95.00% CI of diff.	Significant?	Summary	Adjusted P Value	
Vehicle vs. LPS	-60.01	-76.81 to -43.21	Yes	****	<0.0001	A-B
Vehicle vs. Column C	-31.25	-47.35 to -15.15	Yes	***	0.0003	A-C
LPS vs. Column C	28.76	11.45 to 46.07	Yes	**	0.0014	B-C
**Figure 7B. AT8 fluorescence intensity - Cortex**						

Number of families	1					
Number of comparisons per family	6					
Alpha	0.05					
Tukey's multiple comparisons test	Mean Diff.	95.00% CI of diff.	Significant?	Summary	Adjusted P Value	
Column A vs. Column B	-6768	-8344 to -5192	Yes	****	<0.0001	A-B
Column A vs. Column C	-4331	-5950 to -2713	Yes	****	<0.0001	A-C
Column A vs. Column D	-216.2	-1869 to 1437	No	ns	0.9860	A-D
Column B vs. Column C	2437	942.3 to 3931	Yes	***	0.0003	B-C
Column B vs. Column D	6552	5020 to 8083	Yes	****	<0.0001	B-D
Column C vs. Column D	4115	2539 to 5691	Yes	****	<0.0001	C-D
**Figure 7C. AT8 fluorescence intensity - CA1**						
Number of families	1					
Number of comparisons per family	6					
Alpha	0.05					
Tukey's multiple comparisons test	Mean Diff.	95.00% CI of diff.	Significant?	Summary	Adjusted P Value	
Column A vs. Column B	-557.4	-696.6 to -418.1	Yes	****	<0.0001	A-B
Column A vs. Column C	-359.4	-489.9 to -229	Yes	****	<0.0001	A-C
Column A vs. Column D	-91.03	-228.2 to 46.12	No	ns	0.3083	A-D
Column B vs. Column C	197.9	61.6 to 334.2	Yes	**	0.0016	B-C
Column B vs. Column D	466.3	323.6 to 609.1	Yes	****	<0.0001	B-D
Column C vs. Column D	268.4	134.2 to 402.6	Yes	****	<0.0001	C-D
**Figure 7D. AT8 fluorescence intensity - DG**						
Number of families	1					
Number of comparisons per family	6					
Alpha	0.05					
Tukey's multiple comparisons test	Mean Diff.	95.00% CI of diff.	Significant?	Summary	Adjusted P Value	
Column A vs. Column B	-322.3	-439.3 to -205.2	Yes	****	<0.0001	A-B
Column A vs. Column C	-150.5	-267.5 to -33.44	Yes	**	0.0062	A-C
Column A vs. Column D	-29.81	-149.7 to 90.03	No	ns	0.9139	A-D
Column B vs. Column C	171.8	59.34 to 284.2	Yes	***	0.0008	B-C
Column B vs. Column D	292.4	177.1 to 407.8	Yes	****	<0.0001	B-D
Column C vs. Column D	120.7	5.295 to 236	Yes	*	0.0369	C-D
**Figure 7F. AT100 fluorescence intensity - Cortex**						
Number of families	1					
Number of comparisons per family	6					
Alpha	0.05					
Tukey's multiple comparisons test	Mean Diff.	95.00% CI of diff.	Significant?	Summary	Adjusted P Value	
Column A vs. Column B	-124.3	-172 to -76.63	Yes	****	<0.0001	A-B
Column A vs. Column C	-23.27	-69.69 to 23.15	No	ns	0.5548	A-C
Column A vs. Column D	-5.207	-51.62 to 41.21	No	ns	0.9910	A-D
Column B vs. Column C	101	53.36 to 148.7	Yes	****	<0.0001	B-C
Column B vs. Column D	119.1	71.42 to 166.8	Yes	****	<0.0001	B-D
Column C vs. Column D	18.06	-28.35 to 64.48	No	ns	0.7367	C-D
**Figure 7G. AT100 fluorescence intensity - CA1**						
Number of families	1					
Number of comparisons per family	6					
Alpha	0.05					
Tukey's multiple comparisons test	Mean Diff.	95.00% CI of diff.	Significant?	Summary	Adjusted P Value	
Column A vs. Column B	-100.2	-138.1 to -62.27	Yes	****	<0.0001	A-B
Column A vs. Column C	-40.16	-77.06 to -3.263	Yes	*	0.0276	A-C
Column A vs. Column D	7.005	-30.9 to 44.91	No	ns	0.9620	A-D
Column B vs. Column C	60.02	22.11 to 97.92	Yes	***	0.0005	B-C
Column B vs. Column D	107.2	68.29 to 146.1	Yes	****	<0.0001	B-D
Column C vs. Column D	47.16	9.257 to 85.07	Yes	**	0.0087	C-D
**Figure 7H. AT100 fluorescence intensity - DG**						
Number of families	1					
Number of comparisons per family	6					

Alpha	0.05					
Tukey's multiple comparisons test	Mean Diff.	95.00% CI of diff.	Significant?	Summary	Adjusted P Value	
Column A vs. Column B	-87.52	-128 to -47.07	Yes	****	<0.0001	A-B
Column A vs. Column C	-35.95	-75.33 to 3.421	No	ns	0.0859	A-C
Column A vs. Column D	12.95	-27.51 to 53.4	No	ns	0.8343	A-D
Column B vs. Column C	51.57	11.11 to 92.02	Yes	**	0.0069	B-C
Column B vs. Column D	100.5	58.96 to 142	Yes	****	<0.0001	B-D
Column C vs. Column D	48.9	8.447 to 89.35	Yes	*	0.0114	C-D
**Figure 7J. AT180 fluorescence intensity - Cortex**						
Number of families	1					
Number of comparisons per family	6					
Alpha	0.05					
Tukey's multiple comparisons test	Mean Diff.	95.00% CI of diff.	Significant?	Summary	Adjusted P Value	
Column A vs. Column B	-91.13	-123.3 to -58.96	Yes	****	<0.0001	A-B
Column A vs. Column C	-103.9	-136.1 to -71.77	Yes	****	<0.0001	A-C
Column A vs. Column D	-28.13	-60.3 to 4.049	No	ns	0.1079	A-D
Column B vs. Column C	-12.81	-44.98 to 19.37	No	ns	0.7232	B-C
Column B vs. Column D	63.01	30.83 to 95.18	Yes	****	<0.0001	B-D
Column C vs. Column D	75.82	43.64 to 108	Yes	****	<0.0001	C-D
**Figure 7K. AT180 fluorescence intensity - CA1**						
Number of families	1					
Number of comparisons per family	6					
Alpha	0.05					
Tukey's multiple comparisons test	Mean Diff.	95.00% CI of diff.	Significant?	Summary	Adjusted P Value	
Column A vs. Column B	-80.8	-105.5 to -56.06	Yes	****	<0.0001	A-B
Column A vs. Column C	-51.78	-76.53 to -27.04	Yes	****	<0.0001	A-C
Column A vs. Column D	3.091	-20.63 to 26.81	No	ns	0.9861	A-D
Column B vs. Column C	29.02	4.593 to 53.44	Yes	*	0.0133	B-C
Column B vs. Column D	83.89	60.51 to 107.3	Yes	****	<0.0001	B-D
Column C vs. Column D	54.87	31.49 to 78.26	Yes	****	<0.0001	C-D
**Figure 7L. AT180 fluorescence intensity - DG**						
Number of families	1					
Number of comparisons per family	6					
Alpha	0.05					
Tukey's multiple comparisons test	Mean Diff.	95.00% CI of diff.	Significant?	Summary	Adjusted P Value	
Column A vs. Column B	-72.64	-100.9 to -44.42	Yes	****	<0.0001	A-B
Column A vs. Column C	-61.97	-90.19 to -33.75	Yes	****	<0.0001	A-C
Column A vs. Column D	9.405	-18.16 to 36.97	No	ns	0.8071	A-D
Column B vs. Column C	10.67	-17.55 to 38.89	No	ns	0.7540	B-C
Column B vs. Column D	82.05	54.48 to 109.6	Yes	****	<0.0001	B-D
Column C vs. Column D	71.38	43.81 to 98.95	Yes	****	<0.0001	C-D
**Figure 8B. DYRK1A fluorescence intensity - Cortex**						
Number of families	1					
Number of comparisons per family	6					
Alpha	0.05					
Tukey's multiple comparisons test	Mean Diff.	95.00% CI of diff.	Significant?	Summary	Adjusted P Value	
Column A vs. Column B	-66.14	-95.75 to -36.52	Yes	****	<0.0001	A-B
Column A vs. Column C	-5.937	-34.77 to 22.89	No	ns	0.9486	A-C
Column A vs. Column D	8.778	-20.05 to 37.61	No	ns	0.8540	A-D
Column B vs. Column C	60.2	30.58 to 89.82	Yes	****	<0.0001	B-C
Column B vs. Column D	74.91	45.3 to 104.5	Yes	****	<0.0001	B-D
Column C vs. Column D	14.72	-14.11 to 43.54	No	ns	0.5397	C-D
**Figure 8C. DYRK1A fluorescence intensity - CA1**						
Number of families	1					
Number of comparisons per family	6					
Alpha	0.05					
Tukey's multiple comparisons test	Mean Diff.	95.00% CI of diff.	Significant?	Summary	Adjusted P Value	

Column A vs. Column B	-68.73	-97.58 to -39.88	Yes	****	<0.0001	A-B
Column A vs. Column C	-16.96	-45.04 to 11.12	No	ns	0.3916	A-C
Column A vs. Column D	-23.69	-52.54 to 5.16	No	ns	0.1445	A-D
Column B vs. Column C	51.77	22.92 to 80.62	Yes	****	<0.0001	B-C
Column B vs. Column D	45.04	15.44 to 74.64	Yes	***	0.0009	B-D
Column C vs. Column D	-6.731	-35.58 to 22.12	No	ns	0.9274	C-D
**Figure 8D. DYRK1A fluorescence intensity - DG**						
Number of families	1					
Number of comparisons per family	6					
Alpha	0.05					
Tukey's multiple comparisons test	Mean Diff.	95.00% CI of diff.	Significant?	Summary	Adjusted P Value	
Column A vs. Column B	-72.33	-103.3 to -41.37	Yes	****	<0.0001	A-B
Column A vs. Column C	-21.07	-51.21 to 9.068	No	ns	0.2639	A-C
Column A vs. Column D	4.176	-26.79 to 35.14	No	ns	0.9846	A-D
Column B vs. Column C	51.26	20.3 to 82.22	Yes	***	0.0002	B-C
Column B vs. Column D	76.51	44.74 to 108.3	Yes	****	<0.0001	B-D
Column C vs. Column D	25.25	-5.717 to 56.21	No	ns	0.1491	C-D
**Figure 8F. p-GSK3α/β fluorescence intensity - Cortex**						
Number of families	1					
Number of comparisons per family	6					
Alpha	0.05					
Tukey's multiple comparisons test	Mean Diff.	95.00% CI of diff.	Significant?	Summary	Adjusted P Value	
Column A vs. Column B	-94.2	-130.3 to -58.11	Yes	****	<0.0001	A-B
Column A vs. Column C	-84.38	-121.5 to -47.29	Yes	****	<0.0001	A-C
Column A vs. Column D	8.067	-29.84 to 45.97	No	ns	0.9442	A-D
Column B vs. Column C	9.813	-24.91 to 44.54	No	ns	0.8805	B-C
Column B vs. Column D	102.3	66.67 to 137.9	Yes	****	<0.0001	B-D
Column C vs. Column D	92.45	55.83 to 129.1	Yes	****	<0.0001	C-D
**Figure 8G. p-GSK3α/β fluorescence intensity - CA1**						
Number of families	1					
Number of comparisons per family	6					
Alpha	0.05					
Tukey's multiple comparisons test	Mean Diff.	95.00% CI of diff.	Significant?	Summary	Adjusted P Value	
Column A vs. Column B	-50.5	-73.11 to -27.89	Yes	****	<0.0001	A-B
Column A vs. Column C	-47.14	-68.28 to -25.99	Yes	****	<0.0001	A-C
Column A vs. Column D	9.402	-12.86 to 31.66	No	ns	0.6845	A-D
Column B vs. Column C	3.363	-19.01 to 25.74	No	ns	0.9789	B-C
Column B vs. Column D	59.9	36.46 to 83.34	Yes	****	<0.0001	B-D
Column C vs. Column D	56.54	34.51 to 78.56	Yes	****	<0.0001	C-D
**Figure 8H. p-GSK3α/β fluorescence intensity -DG**						
Number of families	1					
Number of comparisons per family	6					
Alpha	0.05					
Tukey's multiple comparisons test	Mean Diff.	95.00% CI of diff.	Significant?	Summary	Adjusted P Value	
Column A vs. Column B	-54.57	-92.08 to -17.07	Yes	**	0.0015	A-B
Column A vs. Column C	-57.46	-94.97 to -19.95	Yes	***	0.0008	A-C
Column A vs. Column D	-9.478	-47.89 to 28.93	No	ns	0.9158	A-D
Column B vs. Column C	-2.89	-38.93 to 33.15	No	ns	0.9967	B-C
Column B vs. Column D	45.1	8.123 to 82.07	Yes	*	0.0105	B-D
Column C vs. Column D	47.98	11.01 to 84.96	Yes	**	0.0057	C-D
**Figure 9A. Aβ_{40} levels**						
Table Analyzed		Thr181				
Column A		Data Set-A				
vs.		vs.				
Column B		Data Set-B				
Unpaired t test						
P value		<0.0001				

P value summary	****
Significantly different (P < 0.05)?	Yes
One- or two-tailed P value?	Two-tailed
t, df	t=12.85, df=10

Figure 9B. Aβ_{42} levels

Table Analyzed	Thr181
Column A	Data Set-A
vs.	vs.
Column B	Data Set-B
Unpaired t test	
P value	<0.0001
P value summary	****
Significantly different (P < 0.05)?	Yes
One- or two-tailed P value?	Two-tailed
t, df	t=13.75, df=10

Figure 9D. p-Tau (Thr181)

Number of families	1					
Number of comparisons per family	3					
Alpha	0.05					
Tukey's multiple comparisons test	Mean Diff.	95.00% CI of diff.	Significant?	Summary	Adjusted P Value	
Vehicle vs. APPswe	-5.532	-8.755 to -2.309	Yes	***	0.0003	A-B
Vehicle vs. APPswe+Lom	-0.381	-3.529 to 2.767	No	ns	0.9546	A-C
APPswe vs. APPswe+Lom	5.151	1.895 to 8.407	Yes	***	0.0009	B-C

Figure 9F. p-Tau (Ser202/Thr205)

Number of families	1					
Number of comparisons per family	3					
Alpha	0.05					
Tukey's multiple comparisons test	Mean Diff.	95.00% CI of diff.	Significant?	Summary	Adjusted P Value	
Vehicle vs. APPswe	-1.635	-3.122 to -0.1474	Yes	*	0.0277	A-B
Vehicle vs. APPswe+Lom	-1.776	-3.263 to -0.2888	Yes	*	0.0151	A-C
APPswe vs. APPswe+Lom	-0.1414	-1.629 to 1.346	No	ns	0.9720	B-C

Figure 9H. p-Tau (Ser396)

Number of families	1					
Number of comparisons per family	3					
Alpha	0.05					
Tukey's multiple comparisons test	Mean Diff.	95.00% CI of diff.	Significant?	Summary	Adjusted P Value	
Vehicle vs. APPswe	-1.805	-3.103 to -0.5073	Yes	**	0.0039	A-B
Vehicle vs. APPswe+Lom	-0.4076	-1.741 to 0.9262	No	ns	0.7455	A-C
APPswe vs. APPswe+Lom	1.398	0.1298 to 2.665	Yes	*	0.0273	B-C

Supplementary Figure 1A. IL-1β ELISA - Cortex

Table Analyzed	Data 1
Column B	Lom
vs.	vs.
Column A	Veh
Unpaired t test	
P value	0.2500
P value summary	ns
Significantly different (P < 0.05)?	No
One- or two-tailed P value?	Two-tailed
t, df	t=1.200, df=14

Supplementary Figure 1B. IL-1β ELISA - Hippocampus

Table Analyzed	Data 1
Column B	Lom
vs.	vs.
Column A	Veh
Unpaired t test	
P value	0.8413

P value summary	ns
Significantly different (P < 0.05)?	No
One- or two-tailed P value?	Two-tailed
t, df	t=0.2040, df=14

Supplementary Figure 1C. IL-6 ELISA - Cortex

Table Analyzed	Data 1
Column B	Lom
vs.	vs.
Column A	Veh
Unpaired t test	
P value	0.8204
P value summary	ns
Significantly different (P < 0.05)?	No
One- or two-tailed P value?	Two-tailed
t, df	t=0.2314, df=14

Supplementary Figure 1D. IL-6 ELISA - Hippocampus

Table Analyzed	Data 1
Column B	Lom
vs.	vs.
Column A	Veh
Unpaired t test	
P value	0.0835
P value summary	ns
Significantly different (P < 0.05)?	No
One- or two-tailed P value?	Two-tailed
t, df	t=1.864, df=14

Supplementary Figure 2A. IL-1β ELISA – Cortex

Number of families	1					
Number of comparisons per family	3					
Alpha	0.05					
Tukey's multiple comparisons test	Mean Diff.	95.00% CI of diff.	Significant?	Summary	Adjusted P Value	
Column A vs. Column B	-3.986	-5.509 to -2.464	Yes	****	<0.0001	A-B
Column A vs. Column C	-3.096	-4.618 to -1.573	Yes	***	0.0001	A-C
Column B vs. Column C	0.8903	-0.6322 to 2.413	No	ns	0.3232	B-C

Supplementary Figure 2B. IL-1β ELISA – Hippocampus

Number of families	1					
Number of comparisons per family	3					
Alpha	0.05					
Tukey's multiple comparisons test	Mean Diff.	95.00% CI of diff.	Significant?	Summary	Adjusted P Value	
Column A vs. Column B	-10.50	-13.97 to -7.035	Yes	****	<0.0001	A-B
Column A vs. Column C	-5.171	-8.637 to -1.705	Yes	**	0.0032	A-C
Column B vs. Column C	5.330	1.864 to 8.796	Yes	**	0.0024	B-C

Supplementary Figure 2C. IL-6 ELISA – Cortex

Number of families	1					
Number of comparisons per family	3					
Alpha	0.05					
Tukey's multiple comparisons test	Mean Diff.	95.00% CI of diff.	Significant?	Summary	Adjusted P Value	
Column A vs. Column B	-13.63	-18.03 to -9.218	Yes	****	<0.0001	A-B
Column A vs. Column C	-9.197	-13.61 to -4.790	Yes	****	<0.0001	A-C
Column B vs. Column C	4.428	0.02049 to 8.836	Yes	*	0.0488	B-C

Supplementary Figure 2D. IL-6 ELISA - Hippocampus

Number of families	1					
Number of comparisons per family	3					
Alpha	0.05					
Tukey's multiple comparisons test	Mean Diff.	95.00% CI of diff.	Significant?	Summary	Adjusted P Value	
Column A vs. Column B	-22.52	-31.11 to -13.93	Yes	****	<0.0001	A-B
Column A vs. Column C	-10.42	-19.01 to -1.824	Yes	*	0.0159	A-C
Column B vs. Column C	12.10	3.508 to 20.70	Yes	**	0.0052	B-C
